# M/M/Infinity Birth-Death Processes – A Quantitative Representational Framework to Summarize and Explain Phase Singularity and Wavelet Dynamics in Atrial Fibrillation

**DOI:** 10.3389/fphys.2020.616866

**Published:** 2021-01-14

**Authors:** Dhani Dharmaprani, Evan Jenkins, Martin Aguilar, Jing X. Quah, Anandaroop Lahiri, Kathryn Tiver, Lewis Mitchell, Pawel Kuklik, Christian Meyer, Stephan Willems, Richard Clayton, Martyn Nash, Stanley Nattel, Andrew D. McGavigan, Anand N. Ganesan

**Affiliations:** ^1^College of Medicine and Public Health, Flinders University, Adelaide, SA, Australia.; ^2^College of Science and Engineering, Flinders University, Adelaide, SA, Australia; ^3^The Research Center, Montréal Heart Institute and Université de Montréal, Montréal, QC, Canada; ^4^Department of Cardiovascular Medicine, Flinders Medical Centre, Bedford Park, SA, Australia; ^5^School of Mathematical Sciences, University of Adelaide, Adelaide, SA, Australia; ^6^Asklepios Klinik, Hamburg, Germany; ^7^University Medical Centre, Hamburg, Germany; ^8^Insigneo Institute for in silico Medicine, University of Sheffield, Sheffield, United Kingdom; ^9^Bioengineering Institute, University of Auckland, Auckland, New Zealand

**Keywords:** atrial fibrillation, phase singularity, Markov model, birth–death process, wavelet

## Abstract

**Rationale:**

A quantitative framework to summarize and explain the quasi-stationary population dynamics of unstable phase singularities (PS) and wavelets in human atrial fibrillation (AF) is at present lacking. Building on recent evidence showing that the formation and destruction of PS and wavelets in AF can be represented as renewal processes, we sought to establish such a quantitative framework, which could also potentially provide insight into the mechanisms of spontaneous AF termination.

**Objectives:**

Here, we hypothesized that the observed number of PS or wavelets in AF could be governed by a common set of renewal rate constants λ_*f*_ (for PS or wavelet formation) and λ_*d*_ (PS or wavelet destruction), with steady-state population dynamics modeled as an M/M/∞ birth–death process. We further hypothesized that changes to the M/M/∞ birth–death matrix would explain spontaneous AF termination.

**Methods and Results:**

AF was studied in in a multimodality, multispecies study in humans, animal experimental models (rats and sheep) and Ramirez-Nattel-Courtemanche model computer simulations. We demonstrated: (i) that λ_*f*_ and λ_*d*_ can be combined in a Markov M/M/∞ process to accurately model the observed average number and population distribution of PS and wavelets in all systems at different scales of mapping; and (ii) that slowing of the rate constants λ_*f*_ and λ_*d*_ is associated with slower mixing rates of the M/M/∞ birth–death matrix, providing an explanation for spontaneous AF termination.

**Conclusion:**

M/M/∞ birth–death processes provide an accurate quantitative representational architecture to characterize PS and wavelet population dynamics in AF, by providing governing equations to understand the regeneration of PS and wavelets during sustained AF, as well as providing insight into the mechanism of spontaneous AF termination.

## Introduction

Atrial fibrillation (AF), the most common human arrhythmia, is characterized by aperiodic and disorganized electrical activation of the atria (Nattel, 2002). In its clinical spectrum, AF episodes may occur in spontaneously terminating paroxysms lasting for as short as a few seconds at a time, through to the most persistent forms of AF, which may self-sustain for many decades (Michaud and Stevenson, 2018). Despite a century of research, the mechanisms sustaining AF are incompletely understood, and a compact quantitative representational architecture with the capacity to accurately summarize and explain the sustained dynamics of AF is lacking.

Here, we aimed to develop such a parsimonious mathematical representational paradigm with the potential to explain the complex dynamics of AF. We postulated that such a representational structure, if established, should have the following properties: (i) it should be able reframe the complexity of AF dynamics in the form of simple governing equations; (ii) these equations should be able to make predictions that can be tested by experimental observation; (iii) the predictions made by the equations should be accurate in as wide a possible range of experimental AF conditions, and be invariant under transformation of scale. Such a quantitative framework could be an important advance in AF dynamics to facilitate reasoning about the underlying pathobiology of AF.

We reasoned that such a quantitative conceptual paradigm could potentially be established by understanding the properties of unstable reentrant circuits in AF. Unstable reentrant circuits, whose pivots are known as phase singularities (Winfree, 1987) (PS), have been a near universal observation in the past century of AF research, occurring at regions of anatomical and functional conduction block, as well as at the center of rotating reentrant circuits (Garrey, 1914; Moe, 1962; Allessie et al., 1985; Mandapati et al., 2000).

In an earlier study, we demonstrated that the formation and destruction of PS in human and experimental AF could be represented as renewal processes (Dharmaprani et al., 2019). Specifically, we showed that the inter-formation and lifetimes of both short and long-lasting PS in human and experimental AF consistently followed exponential distributions (Dharmaprani et al., 2019). These exponential distributions imply quasi-stationary rates of PS formation and destruction, whose rate constants we defined as λ_*f*_ and λ_*d*_ (Dharmaprani et al., 2019). We confirmed in a systematic review, this hallmark of a renewal process was identifiable in all studies of cardiac fibrillation reporting PS lifetime data (Chen et al., 2000a,Chen et al., 2000b; Rogers, 2004; Kuklik et al., 2017; Child et al., 2018; Christoph et al., 2018), suggesting renewal processes as a potentially universal paradigm to explain PS formation and destruction in cardiac fibrillation (Dharmaprani et al., 2019).

**FIGURE 1:**
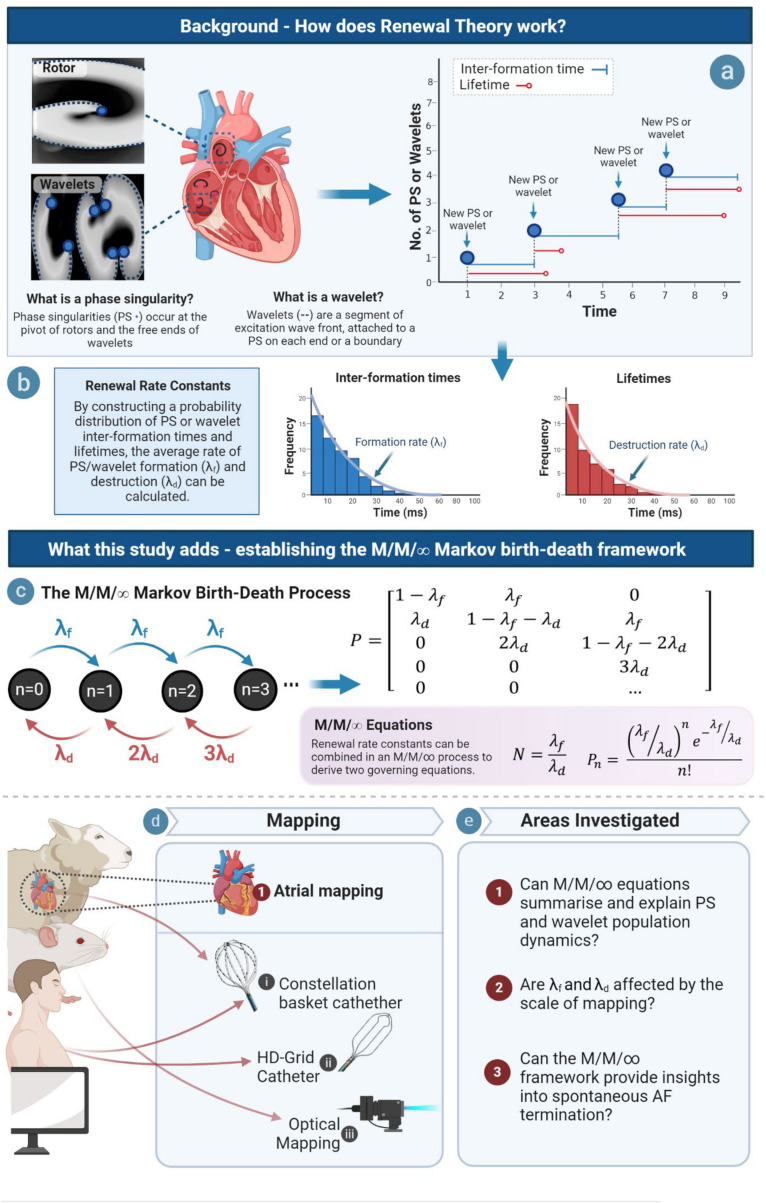
Study overview. **(a)** PS and wavelet lifetimes and inter-formation times are measured, and rate constants of PS/wavelet formation and destruction are obtained by constructing probability distributions of inter-event times **(b)**. Rate of formation (λ_*f*_) gives a measure of how fast PS/wavelets are created, while rate constant of destruction (λ_*d*_) gives a measure of how fast they are destroyed. **(c)** We hypothesized that an M/M/∞ Markov Birth–Death model would provide governing equations of PS/wavelet population, but also potentially provide insights into spontaneous AF termination. An example transition diagram (left) and transition matrix (right) is shown (Supplementary Material S9) **(d)** This hypothesis was investigated across model systems and mapping modalities. **(e)** In order to establish an M/M/∞ framework, three key areas were investigated.

Here, we seek to build on the renewal process paradigm by using the governing parameters established in our previous study, λ_*f*_ and λ_*d*_, to develop a model to understand the dynamics of PS and wavelets at the steady state population level. Given the finding that PS formation and destruction occur at a constant rate, we reasoned that this could be achieved using an M/M/∞ birth–death process; a specific type of continuous time Markov chain in which the population dynamics of a system can be conceptualized as a multi-server queue if arrivals (in the AF case PS or wavelets) occur at a constant renewal rate (λ_*f*_), and each arrival experiences immediate service and is thereby available for destruction (at a constant rate given by λ_*d*_) (Kleinrock, 1976). M/M/∞ birth death processes are characterized by a Markov birth–death transition matrix and well-defined quasi-stationary state equations, and are considered an important foundational concept in probability theory (Figure 1) (Kleinrock, 1976).

In a multispecies and multimodality investigation, we analyzed human, animal experimental and computer simulated AF, hypothesizing the M/M/∞ framework could provide governing equations to explain the steady state average number and population distribution of PS. Due to the intrinsic topological property of PS that connects these circuits to the free ends of wavelets (Winfree, 1987), we further hypothesized that the rate constants λ_*f*_ and λ_*d*_ for PS would be correlated with those for wavelets. Additionally we hypothesized that although λ_*f*_ and λ_*d*_ would vary with coarse graining and decreased field of view (as occurs with human catheter based mapping), that the M/M/∞ governing equations themselves would show the property of scale invariance, and continue to apply at each of these different scales of observation. Finally, we sought to use the M/M/∞ framework to understand the persistence and termination of AF, with the hypothesis that episodes of terminating AF would be distinguished by alterations in spectral properties of the eigenvalues of the birth–death matrix. We specifically reasoned that slowing of the mixing rate at which the quasi-stationary PS birth–death steady state is achieved would be associated with the spontaneous termination of AF, by allowing greater opportunity for the PS population distribution to deviate from the quasi-stationary steady state, and thereby facilitating spontaneous termination. Collectively, this study aimed to establish whether M/M/∞ birth–death processes could be used as a quantitative representational framework to understand how AF self-sustains.

## Materials and Methods

This study tests the hypothesis that the steady state number of rotors and wavelets could be modeled as a Markov M/M/∞ birth–death process, characterized by rate constants λ_*f*_ and λ_*d*_, respectively. Human, animal, and computational models of AF were used to test this hypothesis at different scales and using different mapping modalities. Methods are presented in two parts: (i) ***Part 1*** provides data acquisition and signal processing details; (ii) ***Part 2*** introduces the M/M/∞ birth–death process.

### Part 1: Data Acquisition and Signal Processing

#### Atrial Fibrillation – Computational Simulation, Experimental and Human AF Data

Phase singularities formation and destruction was modeled using a birth–death process in computer simulated AF. Computer simulations were carried out on two-dimensional square grids [Courtemanche-Ramirez-Nattel cell model (Courtemanche et al., 1998), 7 × 6 cm]. Differential equations were solved with a 25 μs time-step, with simulations of up to 5 s. AF epochs were initiated by a standard S1–S2 cross-shock protocol (Supplementary Material S1). The human AF study extends our previously published study (Dharmaprani et al., 2019). The study was a multi-center observational design analyzing electrograms acquired prior to ablation. The inclusion criterion was AF undergoing ablation. Patient participation was by informed consent, with recruitment from Flinders University and Hamburg University. The patient cohort included *n* = 26 patients [age 62 ± 8, 21/26 male (81%), BMI 29 ± 5, non-paroxysmal 18/26 (69%)]. Patient baseline characteristics for the human AF cohort are provided in Supplementary Material S2. Basket catheter recordings were performed as previously (Dharmaprani et al., 2019). 64-electrode basket catheters [Constellation, Boston Scientific, 48 mm (4 mm spacing), 60 mm (5 mm spacing)] were utilized. Unipolar electrogram (1–500 Hz, 2000 Hz sampling) and surface electrocardiogram were obtained in spontaneous or induced AF lasting > 5 min. In a subset of patients, simultaneous basket and grid catheter (Advisor^TM^ -HD grid, Abbott, IL, United States) recordings were obtained, with the HD-grid a 3–3–3 mm catheter with 16 electrodes. During these recordings, the HD grid catheter was placed on the anterior LA, or posterior LA with basket *in situ*. The animal models used included an ovine persistent AF model (mapped with basket catheters) and rat AF model (mapped with optical mapping). Ovine persistent AF was induced via atrial tachypacing for 16 weeks at ≥300 beats per minute as described (Supplementary Material S3) (Dharmaprani et al., 2019). Unipolar electrograms were obtained during electrophysiology study, using 64-electrode Constellation catheters (48 mm). Electrograms were filtered from 30–500 Hz and sampled at 1 kHz for sheep (Supplementary Materials S3, S5). Five-minute or greater recordings from the LA and RA were obtained in AF. Optical mapping in a rat AF model was performed as previously described (Supplementary Material S4) (Dharmaprani et al., 2019). The heart was excised and perfused in Langendorff mode with Krebs solution at 30 ml/min and 37°C. After stabilization and blebbistatin electrical–mechanical decoupling, the heart was loaded with di-4-ANEPPS (Dharmaprani et al., 2019). A charge-coupled device was used to record fluorescence (Dharmaprani et al., 2019) (Supplementary Material S4).

#### Cleaning, Filtering and Signal Processing

**FIGURE 2:**
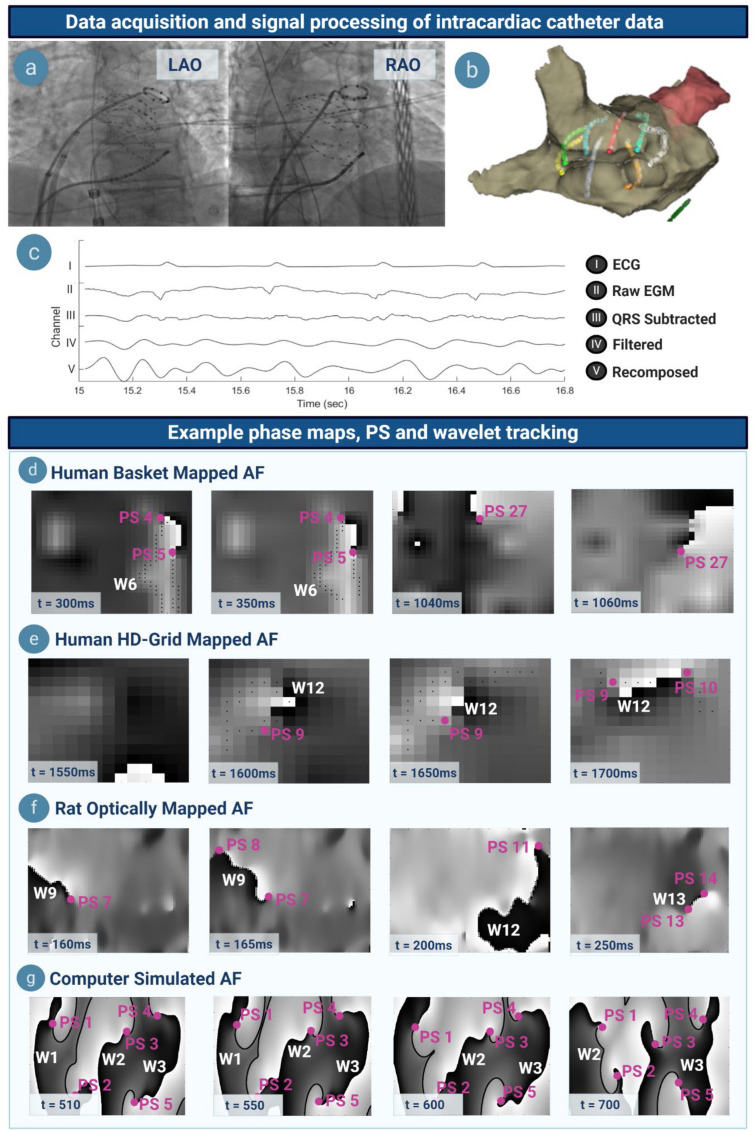
Data acquisition, signal processing, example phase maps and tracking. **(a)** Left anterior oblique (LAO) and right anterior oblique (RAO) projection showing a basket catheter *in situ* in the atrium. **(b)** Three-dimensional visualization of the same basket catheter within the anatomic shell, mapped on Velocity. **(c)** Atrial electrograms undergo QRS subtraction to remove ventricular artifacts, a series of filtering steps, and sinusoidally recomposed. **(d)** Phase maps in basket-mapped human AF, with PS denoted by (∙PS) and wavelets by (W). A wavelet can be seen at *t* = 300–350 ms, whilst a rotor at *t* = 1040–1060 ms. **(e–g)** Phase maps of HD-grid mapped human, optically mapped rat, and computer simulated AF. Note that phase map resolution in catheter-mapped data corresponds to the respective electrode interspacing.

Signal processing was performed as previously (Supplementary Materials S3, S5) (Dharmaprani et al., 2019) (Figure 2). Unipolar electrograms, surface ECG, and 3D data were exported from EnSite Velocity. QRS subtraction was performed (Dharmaprani et al., 2019). Further pre-processing was applied using Butterworth filters applied in forward and reverse mode for sheep and human data (Supplementary Materials S3, S5) (Dharmaprani et al., 2019). Sinusoidal recomposition was applied with dominant frequency used as the wavelet period (Dharmaprani et al., 2019) and phase computed using the Hilbert transform (Dharmaprani et al., 2019). In optically mapped data, a Gaussian kernel (σ = 4) was used to perform lowpass spatiotemporal filtering on the transmembrane potential (Dharmaprani et al., 2019). The Hilbert transform was applied to filtered optically mapped and simulated transmembrane voltages, respectively (Supplementary Material S5).

#### Phase Singularity and Wavelet Detection and Tracking

Phase singularities are topological defects at the pivots of rotors, and at the free ends of wavelets (Figure 1a) (Winfree, 1987). PS were detected using two topological charge methods: (i) a convolutional kernel method (Bray and Wikswo, 2002; Nash et al., 2006; Child et al., 2018) and (ii) the double ring method (Kuklik et al., 2017) (Supplementary Material S7 and Supplementary Figure 23, S16). PS and wavelets were tracked using a previously implemented tracking algorithm (Nash et al., 2006; Bradley et al., 2011; Child et al., 2018; Dharmaprani et al., 2019). New PS were defined as the detection of a PS not falling within the surrounding electrode neighborhood of radius *r* of another PS, and existing for a duration of τ. Primary analysis was performed with τ = 10 ms and *r* = 4 mm in human basket AF, with sensitivity analyses performed for a range of τ and *r* values to validate the consistency. Wavefronts were identified as lines of zero phase, and tracked using graph theory approach as described (Rogers, 2004) (Supplementary Material S7). To determine wavelet and PS lifetime and inter-formation times, a look-up table indexing onset time, offset time and electrode location for each new PS and wavelet detection was created (Dharmaprani et al., 2019).

### Part 2: Study Methodology

#### Establishing Summary Equations of PS and Wavelet Population Dynamics in AF

A birth–death process is a continuous-time Markov chain used to represent the number of entities in a dynamical system (Kleinrock, 1976). An introduction to Markov birth–death processes is provided in Supplementary Materials S8–S10, and Figure 1. In a birth–death process, there are two types of state transitions: (i) ‘births,’ which increase the population size by one, and (ii) ‘deaths’ that reduce the population by one (Figure 1c). In AF, we hypothesized, based on our earlier demonstration that PS and wavelet formation rates (λ_*f*_) and destruction (λ_*d*_) are quasi-stationary (Supplementary Material S9), that the population dynamics of PS and wavelets could be summarized as an M/M/∞ birth–death process. The M/M/∞ is a type of birth–death process in which there is a quasi-stationary rate of arrivals into the system (with renewal rate constant λ_*f*_), and a similarly stable rate of destruction (with renewal rate constant λ_*d*_), that occurs in the scenario that arrivals into the system are immediately available for service. In AF, we reasoned that because as soon as PS and wavelets are formed, they are immediately accessible for potential destruction, we reasoned the M/M/∞ birth–death process could potentially be used to model AF PS population dynamics. A characteristic of M/M/∞ birth–death processes is the presence of a well-defined transition matrix (Supplementary Material S10) that converges to a quasi-stationary steady state population dynamics with fixed, predictable conditional limiting distributions (Van Doorn, 1991). It can be noted that Markov chain theory allows for the state space to be infinite, and any calculations of quasi-stationary states equations, would be exact in the limiting case. However, in this study, the birth–death matrix was estimated using the maximum number of PS/WF tracked states in the birth–death design matrix. From this, two key M/M/∞ equations were used to model: (i) the average number of PS and wavelets, and (ii) the population distribution of PS and wavelets.

For an M/M/∞ birth–death processes, the average number *N* of PS and wavelets may be summarized using the steady-state equation (Kleinrock, 1976):

$$N=\frac{\lambda_{f}}{\lambda_{d}}\;\left(1\right)$$

where λ_*f*_ and λ_*d*_ are the rates of PS/wavelet formation and destruction, respectively.

The second equation summarizes the PS and wavelet population distribution, which gives the steady-state probability (*P*_*n*_) of having a phase singularity or wavelet population size of *n* (Kleinrock, 1976):

$$P_{n}=\frac{{\left(\lambda_{f}/\lambda_{d}\right)}^{n}e^{-\lambda_{f}/\lambda_{d}}}{n!}\;\left(2\right)$$

To test the hypothesis that these equations explain and summarize PS and wavelet population dynamics, λ_*f*_ and λ_*d*_ were obtained as described previously (Dharmaprani et al., 2019) and used to model the number and population distribution of PS/wavelets present in the recording. This modeled prediction was compared to the observed number and population distribution of PS and wavelets.

#### Effect of Mapping Scale on PS and Wavelet Population Dynamics

As the size of the mapped area would influence the number of PS and wavelet formation and destruction events captured, we hypothesized that this would in turn affect the calculated λ_*f*_ and λ_*d*_, relevant to the clinical application of the steady state equations. To test this hypothesis of scale dependence, we investigated PS/wavelet population dynamics and renewal rates in: (i) basket catheter recordings of human persistent AF, (ii) HD-grid recordings of human persistent AF and (iii) computer simulated AF. To gain insight into this effect, decimation was also used in AF computer simulations to investigate how the spatial density of the mapped area affects PS and wavelet formation and destruction processes (Supplementary Material S11). Spatially uniform pixels were selected at progressively coarse-grained spatial densities in simulated AF. Phase between the remaining pixels was interpolated, and PS/wavelet rates and population measured.

#### Insights Into Spontaneous AF Termination

An interesting, yet incompletely understood characteristic of AF is its ability to spontaneously terminate into sinus rhythm (Lin et al., 2017). We hypothesized because termination is a deviation from the quasi-stationary state, the eigenvalue spectrum of the birth–death transition matrix could explain termination (further detail in Supplementary Material S10). The spectral gap, calculated as the difference between the first and second largest eigenvalues, is the key determinant of the rate at which a Markov matrix approaches its quasi-stationary distribution (Boyd et al., 2004).

We specifically reasoned that spontaneous AF termination would likely occur when steady state is reached more slowly, thereby providing a greater opportunity for the process to diverge from the quasi-steady distribution and break the cycle of PS and wavelet regeneration. To test this hypothesis, we examined the eigenvalue spectral gap in computer simulated and human AF, comparing cases where AF spontaneously terminated to cases where it sustained. The spectral gap determines the ‘*mixing rate*’ at which a Markov matrix approaches its quasi-stationary state, and we hypothesized that the spectral gap should be smaller in self-terminating episodes, along with a slower mixing rate (Supplementary Material S10). Mixing rates, λ_*f*_, λ_*d*_ were estimated from epochs of spontaneous AF termination, and compared to epochs of sustained AF.

#### Cross Validation and Sensitivity Analyses

To cross-validate, several sensitivity analyses were conducted. To ensure that study findings were not due to PS/wavelet annotation method, analyses were repeated with a second PS detection method (Kuklik et al., 2017). We also systematically checked the sensitivity of the PS and wavelet tracking algorithm to confirm the study findings, such that PS were required to be present for τ ≥ 5–40 ms, and tracked within a radius *r* (2.83 pixels, 5.67 pixels, 8.49 pixels and 11.31 pixels on the interpolated 29 × 29 pixel grid). Analyses were also performed for long-lasting PS > 150 ms, corresponding to more stable re-entrant activity, to assess if there were differences in the efficacy of the M/M/∞ equations in more sustained reentry. We also reasoned that due to the intrinsic topological connection whereby PS must occur at the free ends of wavelets, that rate constants of wavelet formation and destruction should be linearly correlated to those for PS λ_*f*_ and λ_*d*_.

#### Statistical Analysis

To calculate λ_*f*_ and λ_*d*_, data was fitted to exponential distributions using maximum likelihood (Dharmaprani et al., 2019). Data fitting was performed in Matlab 2017b (Natick, MA, United States) to create a probability distributions for each epoch. Chi-squared (χ^2^) goodness-of-fit tests were used to assess adequacy of distributional fit. Shapiro–Wilk tests were performed to assess normality for reported variables. To assess statistical significance, *t*-tests were used for independent groups, and in non-normally distributed data the non-parametric Mann–Whitney *U* test was utilized. Comparison of predicted vs. directly observed parameters was performed using bivariate Pearson’s correlation and χ^2^ goodness-of-fit tests. Two-tailed *P*-values were considered significant at *P* < 0.05.

## Results

### M/M/∞ Equations and PS and Wavelet Number in Simulated, Experimental and Human AF

**FIGURE 3:**
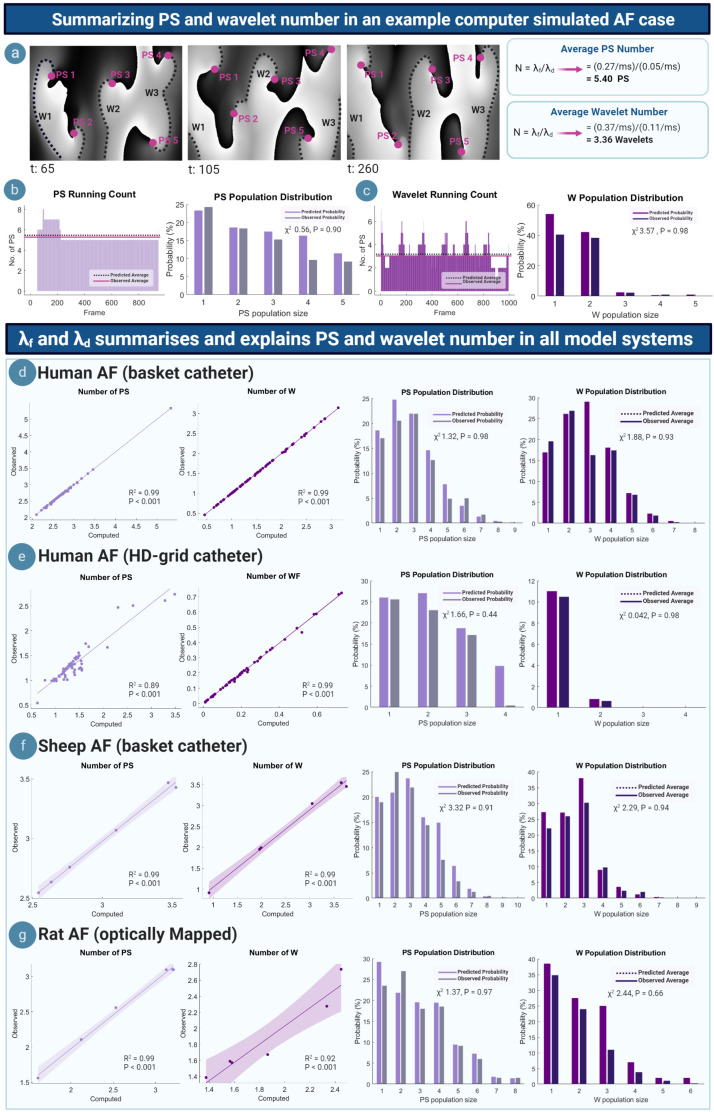
Summarizing and explaining PS and wavelet number. **(a)** Example phase maps from a computer simulated AF case showing the PS/wavelets present at different time steps. Computed average number of PS/wavelets approximate the number observed in the example phase maps. **(b)** Left: running count of PS, showing that the average number of observed PS approximates the computed average number. **(c)** Right: computed and observed population distributions are close matching. **(d)** Left: Scatter plots showing average number of PS and wavelets in human AF (basket catheter mapped). Each point represents the average number of PS or wavelets calculated using equation 1, plotted against the average observed number of PS/wavelets that is seen in a single AF epoch. In all cases of basket -mapped human AF, the average computed and observed number of PS (*R*^2^ = 0.99; *P* < 0.001) and wavelets (*R*^2^ = 0.99; *P* < 0.001) are highly correlated. Computed PS and wavelet population distributions are also well fitted to observed PS (χ_2_ 0.56, *P* = 0.98) and wavelet (χ_2_ 3.57, *P* = 0.93) population distributions. **(e–g)** Similar observations were seen in HD-grid mapped AF, (PS: *R*^2^ = 0.89; *P* < 0.001; wavelets: *R*^2^ = 0.99; *P* < 0.001) (PS: χ_2_ 1.66, *P* = 0.44 wavelet: χ_2_ 0.042, *P* = 0.98), sheep AF (PS: *R*^2^ = 0.99; *P* < 0.001; wavelet: *R*^2^ = 0.99; *P* < 0.001) (PS: χ_2_ 3.32, *P* = 0.91; wavelet: χ_2_ 2.29, *P* = 0.94), rat AF (PS: *R*^2^ = 0.99; *P* < 0.001; wavelet: *R*^2^ = 0.92; *P* < 0.001) (PS: χ_2_ 1.37, *P* = 0.97; wavelet: χ_2_ 2.44, *P* = 0.66).

We tested the ability of the M/M/∞ equations to summarize and explain PS and wavelet dynamics by conducting a multimodality multispecies study comparing the average number and population distribution of PS and wavelets predicted by the M/M/∞ equations. Figure 3a shows phase maps for an example computer simulated AF case (Courtemanche model) (Courtemanche et al., 1998), corresponding to a PS formation rate of λ_*f*_ = 0.27/ms and a PS destruction rate of λ_*d*_ = 0.05/ms. A worked example using the M/M/∞ equation is shown in Figure 3a, giving a computed average number of 5.4 PS. The average wavelet number was also calculated for λ_*f*_ = 0.37/ms and λ_*d*_ = 0.11/ms, giving an average computed number of 3.36 wavelets. Computed PS and wavelets number corresponded to the observed number shown in phase maps (Figure 3a). Figures 3b,c shows the running count (number of PS and wavelets) across time, demonstrating close matching of the average computed and observed number of PS and wavelets. The computed and observed population distributions of PS and wavelets were also close matching, giving χ^2^ values of 0.56 (*P* = 0.91) and 3.57 (*P* = 0.98), respectively, failing to reject the null hypothesis that the distributions are statistically different at the accepted significance level of α = 0.05. This implies a good fit between observed and M/M/∞ predicted population distributions.

All cases of human basket AF showed similar results. The M/M/∞ predicted and observed average number of PS and wavelets were linearly correlated (PS: *R*^2^ = 0.99; *P* < 0.001; wavelets: *R*^2^ = 0.99; *P* < 0.001). M/M/∞ predicted population distributions of PS and wavelets were similar to the observed distributions in all cases, with *P* > 0.05 indicating a good fit to predicted distributions (Figure 3d). Similar findings were seen in all cases of human HD-grid mapped AF (PS: *R*^2^ = 0.89; *P* < 0.00; wavelet: *R*^2^ = 0.99; *P* < 0.001, Figure 3e), all cases of sheep basket catheter-mapped AF (PS: *R*^2^ = 0.99; *P* < 0.001; wavelet: *R*^2^ = 0.99; *P* < 0.001, Figure 3f) and optically mapped rat AF (PS: *R*^2^ = 0.99; *P* < 0.001; wavelet: *R*^2^ = 0.92; *P* < 0.001, Figure 3g). In all model systems, predicted PS population distributions were comparable to observed population distributions (Goodness of fit *P* > 0.05 in all cases, with summary human data presented in Supplementary Table 2 and Supplementary Material S12).

To investigate the relationship between PS and wavelet rates of formation and destruction, the correlation between PS/wavelet λ_*f*_ and λ_*d*_ was studied (Supplementary Material S13). λ_*f*_ and λ_*d*_ for wavelet and PS were correlated (λ_*f*_: *R*^2^ = 0.86; *P* < 0.001; λ_*d*_: *R*^2^ = 0.60; *P* < 0.001), implying λ_*f*_ and λ_*d*_ are a common set of rate constants.

The key M/M/∞ findings were cross validated by assessing the correlation between the predicted and observed PS/wavelet number detected using various tracking parameters (Supplementary Material S14) for the PS/wavelet algorithms (Supplementary Material S16) (number of tracked frames τ and tracking radius *r*). Predicted and observed PS number were linearly correlated for all conditions of the parameters of τ and *r* (Supplementary Figures 7, 8 and Supplementary Material S14).

As the steady state M/M/∞ equations assume λ_*f*_ and λ_*d*_ are stationary (temporally stable) we systematically validated the stationarity of PS and wavelet renewal rates λ_*f*_/λ_*d*_ in all model systems (Supplementary Material S15). In all cases, the autocorrelation of PS inter-formation times approached zero for all non-zero lags, and λ_*f*_ and λ_*d*_ from random short-duration windows converged to long-term λ_*f*_/λ_*d*_, implying temporal stability of these rate constants.

### Effect of Mapping Scale

#### Effect of Mapped Field of View in Computer Simulated AF

**FIGURE 4:**
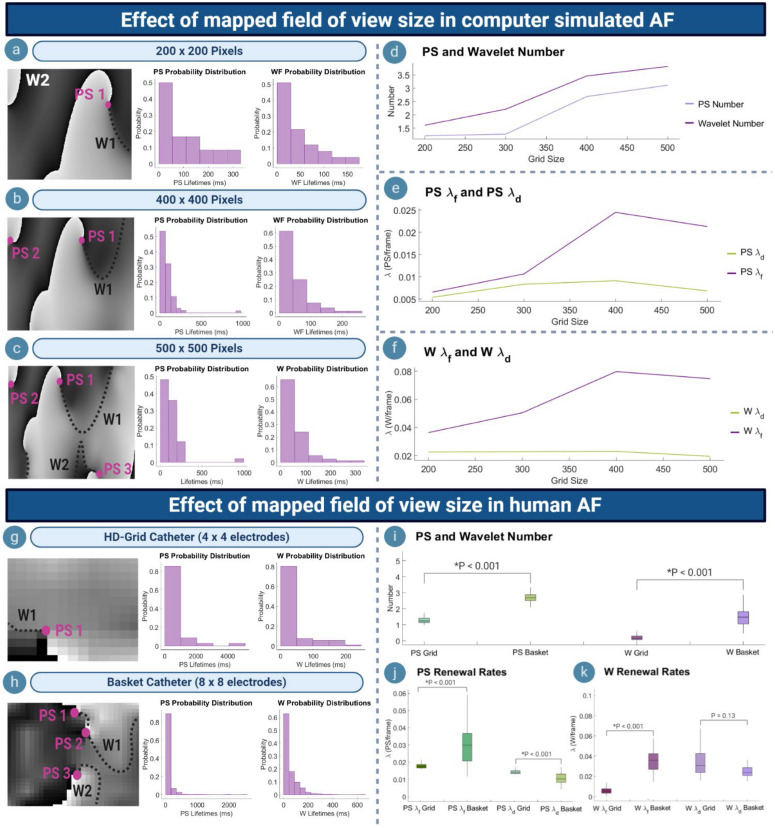
Effect of mapped field of view size. **(a–c)** Phase maps and PS/wavelet lifetime distributions in an example case of computer simulated AF, showing the effect of varying the mapped field size. **(d)** As mapped area increases, more PS/wavelets are captured. **(e)** Increased number of PS captured results in a higher rate of PS formation (λ_*f*_), but consistent PS destruction (λ_*d*_). **(f)** Wavelet λ_*f*_ also increases, but λ_*d*_ remains consistent. **(g,h)** Phase maps and probability distributions of PS/wavelets acquired using HD-grid and basket catheter. **(i)** Basket mapped data with a larger field of view captures a greater number of PS/wavelets. **(j)** PS λ_*f*_ increases for basket catheter data, but λ_*d*_ decreases. **(k)** For wavelets, λ_*f*_ increases when mapped with basket catheter, whilst λ_*d*_ decreases.

Figures 4a–c shows phase maps for various grid-sizes (number of pixels) in computer simulated AF to represent recordings with variously sized fields of view. For each grid size, the corresponding probability distribution of PS/wavelet inter-formation times and lifetimes was most consistent with an exponential distribution, demonstrating consistency of PS formation and destruction as renewal processes at each scale. As the size of the mapped field was increased, the number of PS and wavelets captured also increased (Figure 4d). The increased number of PS and wavelets captured by larger fields of view caused the timings between observed PS formation events to decrease. This resulted in a faster rate of new PS and formations being captured, in turn leading to an aggregate increase in the long-term average rate of PS formation λ_*f*_ (Figure 4e). The long-term average rate of wavelet formation λ_*f*_ also increased in larger grids (Figure 4f). For PS and wavelet destruction events, although the number of events similarly increased with grid size (Figure 4d), the long-term average rate of PS/wavelet destruction λ_*d*_ is more consistent (Figure 4f). It can be noted that for the largest 500 × 500 grid, a slight decrease in PS λ_*f*_ and λ_*d*_ was observed. This is likely due to the long-lasting PS located at the boundary, which is only captured by the 500 × 500 grid (Figure 4c). Consequently, the rate of formation and destruction is slowed slightly. However, overall, the increased number of PS and wavelets detected in larger grids suggests an aggregate increasing trend in λ_*f*_ and λ_*d*_.

#### Effect of Mapped Field of View in Human AF

To further understand the effect of scale dependence on PS formation and destruction rates, λ_*f*_ and λ_*d*_ were calculated for simultaneous basket catheter (8 × 8 electrodes) and HD-grid (4 × 4 electrodes) recordings taken during human persistent AF. Figures 4g,h show example phase maps from the HD-grid catheter recording and the corresponding basket catheter recording of the same human persistent AF epoch. Probability distributions of PS and wavelet inter-formation and lifetimes were most consistent with exponential distributions.

The mean number of PS [1.32, (95% CI, 1.22, 1.42)] and wavelets [0.21, (95% CI, 0.16, 0.25)] captured by the HD-grid catheter was significantly lower than mean the number of PS [2.87, (95% CI, 2.71, 3.04)] and wavelets [1.51, (95% CI, 1.38, 1.65)] captured by the basket catheter (HD-grid: *P* < 0.00; Basket: *P* < 0.001) (Figure 4i).

Differences in rate constants were also seen between the two mapping catheters. The mean PS λ_*f*_ for HD-grid recordings was 0.002/ms (95% CI, 0.001, 0.003) and mean PS λ_*d*_ 0.001/ms (95% CI, 0.001, 0.002). For basket catheter recordings the mean PS λ_*f*_was 0.030/ms (95% CI, 0.027, 0.033) and mean PS λ_*d*_ 0.012/ms (95% CI, 0.010, 0.013) (Figure 4j). For wavelets, the mean λ_*f*_ and λ_*d*_ measured by the HD-grid was 0.006/ms (95% CI, 0.005, 0.007) and 0.036/ms (95% CI, 0.032, 0.039), respectively, and for basket catheter recordings mean λ_*f*_ and λ_*d*_ was 0.037/ms (95% CI, 0.034, 0.39) and 0.029/ms (95% CI, 0.025, 0.033), respectively (Figure 4k). The HD-grid captured a significantly slower PS/wavelet λ_*f*_ when compared to the basket catheter (HD-grid: *P* < 0.001; Basket: *P* < 0.001). However, λ_*d*_ was slightly higher for PS when measured using the HD-grid versus basket catheter (*P* < 0.001), but not wavelet λ_*d*_ (*P* = 0.013) (Figures 4j,k). These findings were similar to those for computer simulated AF.

#### Effect of Mapping Density

**FIGURE 5:**
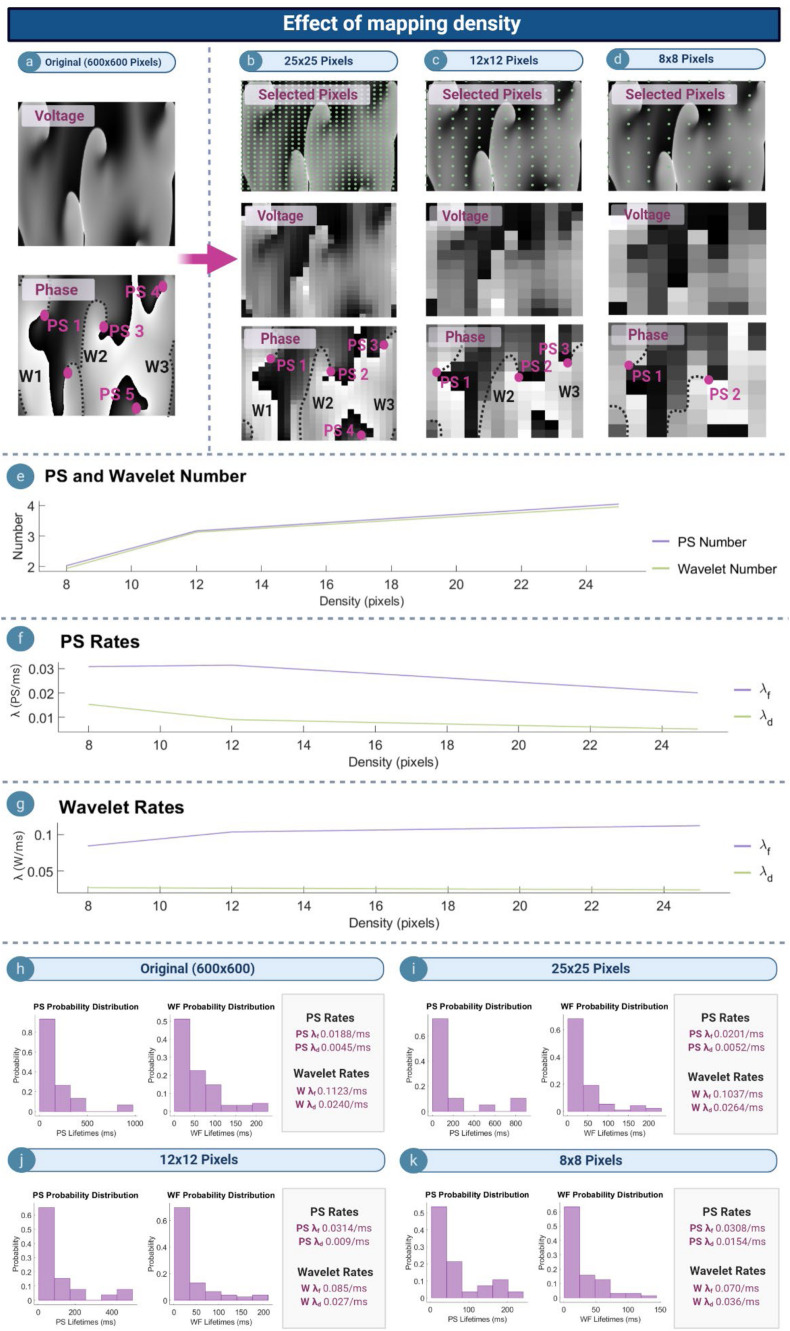
Effect of mapping density. **(a)** Voltage and phase map of the original 600 × 600 pixel computed simulated AF grid. **(b–d)** Top row: selected pixels indicated by the green dot (∙), and used to construct lower resolution grids of 25 × 25, 12 × 12, and 8 × 8 pixels. Corresponding voltage/phase maps are shown **(e)** As mapping density increases, more PS/wavelets are detected. **(f,g)** with increasing mapping density PS λ_*f*_ and λ_*d*_ decreases, and wavelet λ_*f*_ increases whilst λ_*d*_ decreases. **(h–k)** Probability distribution of PS and wavelet lifetimes are exponential for all densities, but λ_*f*_ and λ_*d*_ change.

Figures 5a–d depict example voltage and phase maps for a computer simulated AF. The original undecimated data (600 × 600 pixels) is shown in Figure 5a, with the PS/wavelets detected shown on the respective phase map. As the spatial density of the mapped area increased, the number of PS detected also increased (Figure 5e), leading to a decrease in λ_*f*_ and λ_*d*_ (Figure 5f). This suggests that PS captured with higher density grids are present for a longer length of time as reflected in the probability distributions (Figures 5h–k), and that new PS formations are captured more quickly. In contrast, λ_*f*_ for wavelets increased as the mapping density decreases, whilst λ_*d*_ increased (Figure 5g). This suggests that wavelets captured may last for a longer length of time with increasing grid size.

### Insights Into Spontaneous AF Termination

**FIGURE 6:**
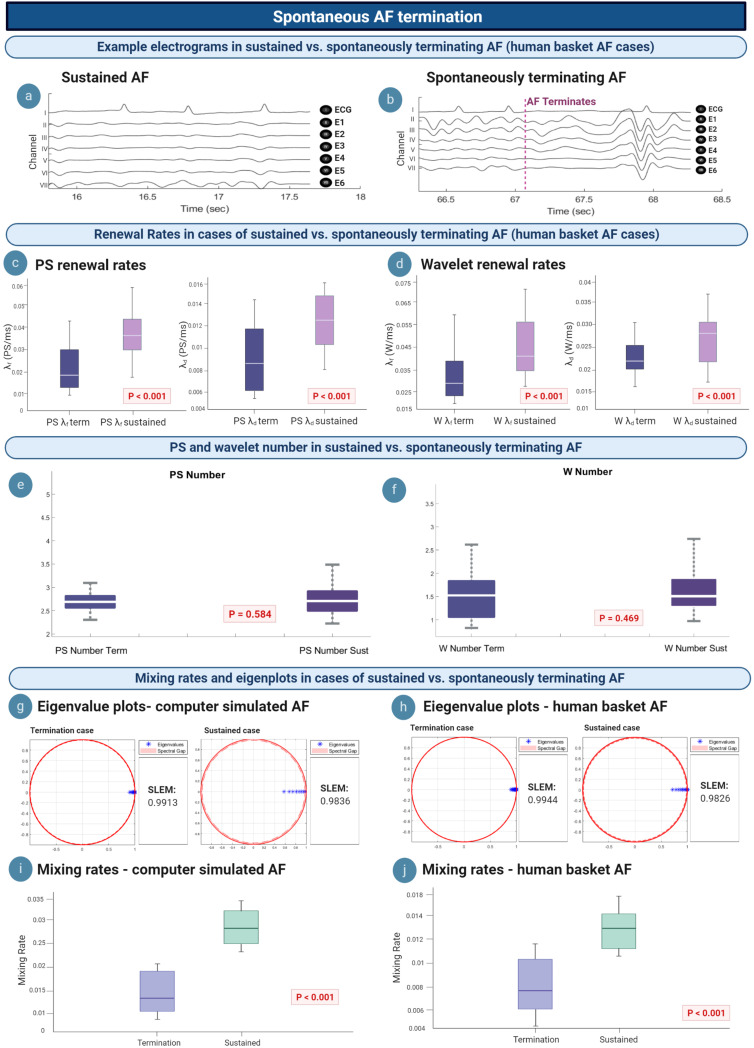
Insights into spontaneous termination. **(a,b)** Example electrograms are shown, with AF terminating at ∼67 s. **(c,d)** λ_*f*_ and λ_*d*_ lowers in cases of spontaneous AF termination, suggesting a slowing in the rate of PS/wavelet regeneration. **(e,f)** Average PS and wavelet number in sustained vs. terminating AF episodes are not statistically different. **(g,i)** Eigenvalue plots for sustained AF and spontaneously terminating AF cases in computer simulated and human AF. In both model systems, termination cases demonstrated a larger value for the second largest eigenvalue modulus (SLEM), which lead to smaller spectral gaps and slower mixing rates. These changes to the eigenvalue transition matrix likely reflect a deviation from steady state, which promotes termination to occur. **(h,j)** In both computer simulated and human AF, mixing rates decrease in cases of spontaneous AF termination (*P* < 0.001).

We hypothesized that we could gain insight into the mechanism of AF termination via understanding differences in the M/M/∞ birth–death matrix between AF epochs that spontaneously terminated and those that sustained. AF termination was studied in *n* = 36 epochs of termination [mean epoch length 88.048 s (95% CI, 70.029, 106.14)], compared to *n* = 56 epochs of sustained AF lasting at least 5 min. Figure 6a shows intracardiac electrograms from a single example epoch of sustained AF, whilst Figure 6b electrograms for an example terminating AF epoch.

The mean PS λ_*f*_ was 0.026/ms (95% CI, 0.023, 0.030) for spontaneous termination and 0.035/ms (95% CI, 0.031, 0.039) for sustained cases. All epochs of spontaneous human AF termination showed a statistically significant difference in PS λ_*f*_ when compared to sustained human AF epochs (*P* < 0.001) (Figure 6c, left). Similar trends were seen for PS λ_*d*_, with spontaneous termination corresponding to a mean of 0.009/ms (95% CI, 0.008, 0.010) and 0.013/ms (95% CI, 0.012, 0.014) in sustained human AF. A statistically significant difference was also seen for PS λ_*d*_ between all terminating versus sustained epochs (*P* < 0.001) (Figure 6c, right). This suggests a slowing of the rate of PS formation and destruction prior to spontaneous AF termination.

The mean wavelet λ_*f*_ was 0.032/ms (95% CI, 0.029, 0.035) and λ_*d*_ 0.022/ms (95% CI, 0.021, 0.024) for epochs showing spontaneous termination. In sustained AF epochs, mean wavelet λ_*f*_ was 0.040/ms (95% CI, 0.036, 0.044) and λ_*d*_ 0.027/ms (95% CI, 0.025, 0.030) (Figure 6d, left). Differences between wavelet λ_*f*_ and λ_*d*_ between sustained and terminating cases was statistically significant (λ_*f*_: *P* < 0.001; λ_*d*_: *P* < 0.001) (Figure 6d, right).

It can be noted that although PS and wavelet λ_*f*_ and λ_*d*_ lowered in cases of AF termination, there was no statistically significant difference between the average number of PS and wavelets in sustained versus terminating episodes of AF (Figures 6e,f). The mean PS number in sustained AF cases was 2.74 (95% CI, 2.62, 2.85) and 2.81 in terminating cases (95% CI, 2.59, 3.03), which was not statistically significant (*P* = 0.584). Similar results were seen for the average number of wavelets, [sustained: 1.62 (95% CI, 1.43, 1.82); terminating: 1.52 (95% CI, 1.31, 1.73)] which were also not statistically significant (*P* = 0.469). We reasoned this lack of difference may be due to the fact that the average PS and WF number is calculated throughout the whole epoch rather than at a specific moment, and since spontaneous AF termination happens rather abruptly, the change in PS and WF number would only occur in the very few moments before termination.

We further reasoned that rather than the average number itself, it may be deviations from the steady state population distribution that differentiates terminating episodes. When analyzing the eigenvalue spectrum of the Markov birth–death transition matrix in computer simulated AF, the second largest eigenvalue modulus (SLEM) of termination cases [mean 0.9839 (95% CI, 0.9668, 1.000)] was consistently higher than for sustained AF cases [mean 0.9771 (95% CI, 0.9558, 0.9919)] (*P* < 0.001) (Figure 6g). This led to smaller spectral gaps, and therefore slower mixing rates for spontaneous AF termination cases. Specifically, the mean mixing rate in computer simulated AF was 0.016 (95% CI, 0.001, 0.034) for spontaneous termination and 0.032 (95% CI, 0.018, 0.045) for sustained cases (*P* < 0.001) (Figure 6i).

Similar results were seen for human AF. The SLEM of termination cases [mean 0.8815 (95% CI, 0.7746, 0.9885)] was consistently higher than for sustained AF cases [mean 0.7826 (95% CI, 0.6279, 0.9373)] (*P* < 0.001) (Figure 6h). All epochs of spontaneous human AF termination showed a significantly slower mixing rate when compared to sustained human AF (*P* < 0.001) (Figure 6j). In human basket AF, the mean mixing rate was also slower in cases of spontaneous AF termination [mean 0.008 (095% CI, 0.007, 0.009)] versus sustained AF [mean 0.013 (095% CI, 0.012, 0.015)].

## Discussion

Despite a century of research, the complex and turbulent dynamics of AF remain incompletely understood. The objective of the current study was to develop an ontologically parsimonious mathematical representation of AF dynamics. We reasoned that because of the intrinsically disaggregated nature of wave propagation in AF across the atrial myocardium, over time PS and wavelet formation and destruction inter-event times would converge to exponential distributions associated with stable rate constants λ_*f*_ and λ_*d*_. By analogy with queueing systems in probability theory, we hypothesized that λ_*f*_ and λ_*d*_ would thus be fundamental rate parameters that could be combined in an M/M/∞ birth–death model to summarize and understand AF dynamics.

We investigated this hypothesis in a multispecies, multimodality study of AF and demonstrated that:

i. λ_*f*_ and λ_*d*_ can be combined using an M/M/∞ birth death process to accurately model the quasi-stationary population dynamics of PS and wavelets in AF.
ii. Wavelet and PS λ_*f*_ and λ_*d*_ are highly correlated in keeping with the topological connection between these two forms of propagation.
iii. Rate constants λ_*f*_ and λ_*d*_ are determined by the field of view of AF mapping, both in terms of the size of the mapped field and the scale effect of coarse graining the system via mapping AF at reduced electrode densities, as is performed in clinical settings. However, the operation of the M/M/∞ birth–death process itself is invariant under scale transformation.
iv. Rate constants λ_*f*_ and λ_*d*_ are slowed in AF cases with spontaneous termination, leading to a reduced spectral gap and slower mixing rate of the M/M/∞ birth–death Markov matrix, potentially providing an explanation for spontaneous AF termination.

These findings suggest the M/M/∞ birth–death process can be used to provide a new quantitative representational framework to understand PS and wavelet population dynamics in AF. We replicated this finding in a range of AF models in different species, using different mapping modalities and densities, to provide evidence of generality. It should be noted an exponential distribution of PS lifetimes has been a consistent observation in both AF and VF (Chen et al., 2000a,Chen et al., 2000b; Rogers, 2004; Kuklik et al., 2017; Child et al., 2018; Christoph et al., 2018), suggesting that the M/M/∞ approach should be readily replicated. The consistency of the M/M/∞ framework over different densities and with coarse-graining suggests that although AF dynamic behavior may appear quite different over various mapping resolutions and fields of view, the underlying fibrillatory process is internally consistent at each level of observation, such that λ_*f*_ and λ_*d*_ measured at any particular observational level give rise to dynamics internally consistent with an M/M/∞ birth–death process operating at the same level.

### What This Study Adds to the Renewal Theory Paradigm

In our previous study, we demonstrated that PS formation and destruction could be represented as renewal processes, characterized by rate constants λ_*f*_ and λ_*d*_ (Dharmaprani et al., 2019). We demonstrated that this applied to various model systems (humans, rat, sheep and computer simulation) and across mapping modalities. Although this work allowed us to quantify λ_*f*_ and λ_*d*_, the previous study did not use these rate constants to develop governing mathematical equations to quantify the population dynamics of PS and wavelets, or study how λ_*f*_ and λ_*d*_ changes under various conditions such as varying mapped fields of view, and in episodes of sustained versus terminating AF. Collectively, the results of the current study extends our previous work by finding a way to utilize λ_*f*_ and λ_*d*_ in an M/M/∞ birth–death process, hence providing a new quantitative representational framework to help further understand AF dynamics.

### Important Considerations in Applying the M/M/∞ Approach

In considering the results of the current study, a number of important issues are worthy of consideration. The first is whether or not the results could have arisen purely by methodological experimental error in signal acquisition, or processing. Several studies have suggested a potential for error in electrogram-based detection of PS with basket catheters (Roney et al., 2017; Martinez-Mateu et al., 2018). We would agree a degree of error is certainly possible that with basket catheter based assessment of PS, which would affect the precision of rate constant estimates for λ_*f*_ and λ_*d*_.

However, several factors would suggest that experimental error alone would be insufficient explain the consistent observation of exponential distributions and the accuracy of the birth–death equations. The first is that an exponential distribution of phase singularity and rotor lifetimes is a consistent finding throughout the history of AF research. The Jalifé laboratory was the first to report an exponential shaped distribution of PS lifetimes in the classic cholinergic sheep model of AF, where PS detection was based on optically mapped data (Chen et al., 2000a). The same pattern was confirmed by the same group in ventricular fibrillation (Chen et al., 2000b). Subsequently, epicardial distributions of rotor lifetimes were observed in careful epicardial recordings, as well as simulations (Kuklik et al., 2017; Schlemmer et al., 2018). We, and others, have observed the same pattern in basket catheter data (Child et al., 2018; Dharmaprani et al., 2019). In our study, we performed we performed careful analyses of PS inter-formation and lifetimes in multiple settings, including simulations, optical mapping, and human data. We undertook multiple steps to minimize the methodological error, including the use of multiple PS detection algorithms, applied with a range of parameters, in multiple systems. It remains possible that there could be some degree of experimental error in our findings. However, it is implausible that the internal consistency of our findings, in conjunction with the consistency of our results with the data has accrued over several decades, could arise purely by chance.

A second important factor to consider in assessing the M/M/∞ framework is to understand that this representational architecture is to understand its logical derivation from the fundamental properties of AF. The fundamental property that separates AF from other atrial arrhythmias is disaggregated electrical activity in the atrium, in both space and time. The intrinsic turbulence of AF as an arrhythmia would suggest that the formation and destruction of PS should be statistically independent events. The exponential distribution of PS inter-formation times, and PS lifetimes, arises as a consequence of this statistical independence, and is a standard finding of probability theory (Ross, 2014). The M/M/∞ equations thus arise as a representational architecture based on this framework. Its consistence concordance with experimentally derived data occurs as a consequence of the intrinsic statistical independence of PS formation and destruction events that are separated in space and time during the disorganized, disaggregated nature of AF.

A third factor in understanding the findings of the current paper is the importance of considering the scale of observation. In our study, we have shown that the M/M/∞ representational architecture can apply at different scales of observation, created by decimation and coarse-graining the observation of AF. We have shown that the rate constants λ_*f*_ and λ_*d*_ are different at each of these scales, but that the equations still apply in each experimental system. This finding has particular relevance to understanding basket catheter data acquired in human, and something that has not previously been considered as an issue before. It is clear based on simulation studies that the under-sampling of AF caused by catheter-based sampling of atrial activity in fibrillation will clearly lead to differences in the nature of PS that are detected (Roney et al., 2017). However, the results of the current study may potentially suggest an alternative interpretation for catheter-based mapping data. We find that that PS and wavefront data from basket- and grid-catheter followed exponential distributions, conformant to the M/M/∞ equations at each specific scale of observation. In a sense, then, an analogy to potentially understand basket catheter and grid data is to consider these as coarse-grained sub-sampling of the fibrillatory process. An analogy to understand this could be the way buoys spaced many kilometers apart in the sea are used to provide information on macro-aggregate phenomena such as ocean currents, but lack the resolution to distinguish very local waves and ripples. Analogously, it is clear that although aggregate phenomena such PS and waves observed at the level of basket, epicardial plaque and grid catheters are quite different to those observed via optical mapping due to differences in spatial resolution, it is clear that the M/M/∞ equations may characterize these phenomena in fibrillation with internal consistency at each of these levels. The reason for this consistency is the statistical independence of PS and wavelet formation and destruction due to the disaggregated, turbulent nature of AF.

### The M/M/∞ Framework as a Parsimonious Representational Paradigm

The M/M/∞ birth–death process is a scientifically attractive conceptual representation of AF dynamics. The equations of the M/M/∞ process are well characterized in probability theory, providing an extensive theoretical framework. Additionally, the framework is also parsimonious, in that the complexity of AF dynamics is shown to be compactly represented by two simple parameters λ_*f*_ and λ_*d*_. This simplicity likely underlies why the M/M/∞ paradigm appears to accurately model PS and wavelet dynamics in a range of model systems. Although these parameters adjust according to the field of view, density of mapping and AF physiology, this consistent model accuracy suggests the M/M/∞ concept itself has the property of invariance under scale.

A key reason the M/M/∞ conceptual paradigm could be quite powerful is that it potentially allows challenging problems within the field to be reframed in quantitative terms, facilitating reasoning about the underlying biology. One such example discussed here is spontaneous termination. The spontaneous termination of AF is a common clinical occurrence that is currently incompletely understood (Kneller et al., 2005; Lin et al., 2017). A common observation prior to termination has been the existence of more organized activity in the pre-termination period (Alcaraz and Rieta, 2009; Thanigaimani et al., 2017). Descriptively, termination has been explained as occurring as a consequence of the final beats of AF occur prior to termination being mediated by the drift of wavelets and PS into the non-conducting atrio-ventricular valves (Allessie et al., 1985; Kneller et al., 2005). Here, we find that a potential explanation for the termination of AF may be differences in the rate of mixing of the birth–death M/M/∞ transition matrix. We find that the rates of PS formation and destruction are consistently slower in AF episodes that terminate compared to those that are sustained, leading to a decreased eigenvalue spectral gap of the birth–death matrix, and slower rate of mixing toward the stationary distribution level. This finding would explain termination as occurring due to the slower rate of return to the quasi-stationary state. This could potentially allow longer stochastic deviations from the equilibrium level of PS to occur, providing a longer window for spontaneous termination to occur.

An important point to recognize is that we would consider the M/M/∞ birth death process a model, or quantitative representation for the dynamics of AF. Although this model appears stable insofar as it provides accurate representations of PS and wavelet behavior, we suggest that this model could potentially be considered as the starting point for a new way of investigating and understanding AF. Much in the way that many scientific conceptual paradigms have been improved by explaining target phenomena outside the frame of reference of particular model abstractions, it is likely that deviations from the M/M/∞ model will provide additional mechanistic and clinical insights, allowing the model to be refined and adapted to particular constraints or scenarios beyond those directly examined. Having said this, to date no such counterexamples have been identified. It is possible the equations may emerge to be considered as idealized governing laws or boundary conditions in AF dynamics.

An interesting issue to consider is why these simple rate constants repeatedly arise. At its core, AF can be considered as a form of chaos, with intrinsically disaggregated and turbulent dynamical behavior arising as a consequence of the intrinsic non-linearity of the atrial myocardium. This non-linearity arises because of the inhomogeneity of individual excitability of atrial myocytes along with the network effects of inhomogeneity due to variability of micro-architecture, and substrate fibrosis. This functional and structural inhomogeneity can be considered as the fundamental substrate for disorganized behavior. The key reason the M/M/∞ pattern repeatedly emerges may be due to the intrinsically turbulent and aperiodic nature of electrical wave propagation that is the defining feature of AF.

### How the M/M/∞ Approach Relates to Contemporary Theories of AF Dynamics

Our findings may contribute to the understanding of the multiple wavelet and rotor theories. The multiple wavelet theory postulates that AF self-sustains by a random process of wavelets moving around the atrium (Moe and Abildskov, 1959). Although the average number of wavelets observed in the atrium has been described experimentally in a number of studies (Allessie et al., 1985; Lee et al., 2015), a quantitative explanation for this number has previously not been provided. The M/M/∞ process provides a novel explanation for this observed average number as arising as the quasi-stationary state arising as a consequence of the interplay between wavelet formation and destruction rates. Importantly, the M/M/∞ representation of AF would differ from classical descriptions of the multiple wavelet theory by suggesting that this quasi-stationary steady state is a predictable and stable phenomenon.

The M/M/∞ birth–death process model also makes a potentially important contribution to the rotor theory. According to this theory, AF is sustained by rapid local re-entrant drivers, with new wavebreaks forming PS at locations of anatomical and functional conduction block (Jalife et al., 2002). The findings add to understanding of the rotor theory by developing a quantitative model to understand PS regeneration. It makes the observation that PS dynamics are defined by statistically definable processes. The M/M/∞ birth–death conceptualization of AF thus potentially provides a bridge between the conventional characterization of the rotor and multiple wavelet theories of AF.

The study findings may have important mechanistic and clinical implications. Mechanistically, the M/M/∞ birth–death process provides compact governing equations to summarize and predict the PS and wavelet population dynamics, providing a novel conceptual framework to understand the underlying mechanisms sustaining AF. This could be used to understand the effects of different AF disease pathophysiology, local microarchitectural effects, or disease substrates, or the effect of potential treatments. It is likely each of the above would lead to alterations in the M/M/∞ constants as a way of mediating their particular effects. The universality of the M/M/∞ constants provides a novel way for the effects of these changes under different experimental conditions to have their overall effect on fibrillatory dynamics precisely quantified. This new conceptual paradigm could potentially allow similarities and differences between these conditions to be understood much more clearly. Clinically, it is clear the M/M/∞ equations are able to model PS and wavelet formation when measured at the typical electrode densities and fields of view during catheter level observation. This would enable the same approach to be applied to compare AF from different patient populations, or patients with different burdens of disease, provided mapping is consistent. The association of these rate constants with the spontaneous termination of AF would suggest that measurement in clinical settings could provide insight into the likely persistence of AF. This may provide new ways to classify AF patients beyond the binary paroxysmal and persistent paradigm. It is likely that although the rate constants were temporally stable over the periods of experimental observation, that changes could occur in longer term follow-up. Longitudinal assessment could potentially provide new physiological approaches to AF classification. In clinical ablation, real-time measurement of the rate constants could potentially be combined with existing or novel ablation strategies to determine treatment effect. If rate slowing is observed, it could imply that the effect of a particular ablation strategy may be having impact on overall dynamics. An additional intriguing possibility is combining local measurement of these rate constants with strategies for detection of potential drivers, potentially providing a means of localization of key regions sustaining AF within the atrium.

### Limitations and Future Directions

As this study primarily investigates PS and wavelet dynamics in AF, the role of other potentially important adjunctive theories such as endo-epicardial dissociation and focal discharges could not be addressed with the experimental data available to us. Our study is based upon the notion of phase transformation, and we acknowledge that while this is a widely utilized approach, it is not the only approach to understand AF dynamics, and that some have investigators have identified that phase singularity identification may be unable to distinguish scenarios of conduction block and rotational activation (Podziemski et al., 2018). We further acknowledge that not all researchers in the field agree with the notion of phase transformation and the study of phase singularity dynamics in AF (Podziemski et al., 2018). We also note that in the electrogram-based component of the current study that sinusoidal recomposition was used for phase reconstruction, and recognize that other approaches such as linear interpolation of phase between activation times in the unipolar electrogram have been suggested as important alternative approaches to phase recomposition (Zeemering et al., 2020). Although this approach was not utilized in this current investigation, we would anticipate it could also potentially lead to PS dynamics that could be modeled with an M/M/infinity process, as we found that M/M/infinity approaches were effective in the optically mapped and simulated AF data where transmembrane voltage was able to be directly ascertained. Furthermore, as this study did not differentiate between wavelets attached to rotors versus free wavelets, the relative contributions of both events were not quantified.

However, applying the framework presented to such scenarios may be an important area for future work to provide new insights into the AF mechanism. An important consideration in applying the findings of the current study is that we make no claim that this approach is superior to other approaches or conceptual theories of AF, only that this approach may potentially provide a quantitative framework to quantify the dynamics of various pattern phenomena (PS, wavefronts), that are observed during AF.

Future studies are required to address the relationship between M/M/∞ equations and different clinical substrates of AF. Features that could be examined include the relationship between these equations and variant ablation strategies, pulmonary vein behavior, voltage characterization in the atrium, as well as spatial characterization of the rate constants throughout the human atrium (Quah et al., 2020). This will be important to determine the practical utility of the governing equations provided by the M/M/∞ birth–death process.

## Conclusion

M/M/∞ birth–death processes provide a novel quantitative representational framework to conceptualize and understand PS and wavelet population dynamics in AF. This conceptual paradigm has been shown to apply in all forms of AF studied, at a variety of different scales and densities of mapping, providing opportunities for clinical application. Finally, the spectral properties of the birth–death matrix potentially identify a new way to understand the process of AF termination, suggesting termination of AF may occur due to differences in the mixing rate of the M/M/∞ birth–death matrix. M/M/∞ birth–death processes may thus potentially provide a powerful representational architecture to gain insight into the pathobiology of AF.

## Data Availability Statement

The raw data supporting the conclusions of this article will be made available by the authors, without undue reservation.

## Ethics Statement

The studies involving human participants were reviewed and approved by Southern Adelaide Local Health Network Human Research Ethics Committee. The patients/participants provided their written informed consent to participate in this study. The animal study was reviewed and approved by South Australian Health and Medical Research Animal Ethics Committee.

## Author Contributions

DD contributed to the analysis and manuscript draft. EJ contributed to the analysis. MA contributed to the modeling and editorial input. AL contributed to the data collection. JQ contributed to the data collection. KT contributed to the data collection and editorial input. PK, CM, and SW contributed to the data collection and editorial input. RC and MN contributed to the editorial input and computer code. SN contributed to the editorial input. AM contributed to the data collection and editorial input. AG contributed to the concept, study design, data collection, and editorial supervision. All authors contributed to the article and approved the submitted version.

## Conflict of Interest

The authors declare that the research was conducted in the absence of any commercial or financial relationships that could be construed as a potential conflict of interest.
