# Supplementary material for: M/M/Infinity Birth-Death Processes – A Quantitative Representational Framework to Summarize and Explain Phase Singularity and Wavelet Dynamics in Atrial Fibrillation
Source: Front Physiol. 2021 Jan 14;11:616866. doi: 10.3389/fphys.2020.616866 (PMC7841497; doi:10.3389/fphys.2020.616866)
Supplement: Supplementary file 1 [file Data_Sheet_1.pdf]

SUPPLEMENTAL MATERIAL

M/M/**infinity** birth-death processes - a quantitative approach to summarise and explain phase singularity and wavelet dynamics in atrial fibrillation

Dhani Dharmaprani, Evan Jenkins, Martin Aguilar, Jing Xian Quah, Lewis Mitchell, Pawel Kuklik, Christian Meyer, Stephan Willems, Richard Clayton, Martyn Nash, Stanley Nattel, Andrew D McGavigan, Anand N. Ganesan

Correspondence:

Dr Anand Ganesan
College of Medicine and Public Health
Flinders University
Flinders Drive, Bedford Park SA 5042
AUSTRALIA
anand.ganesan@flinders.edu.au

CONTENTS

Part 1: Supplementary methods 3
S1 Computer Simulations of Atrial Fibrillation 4
S2 Patient Baseline Characteristics- AF Cohort 5
S3 Sheep Atrial Fibrillation Model 5
S4 Rat AF – Optical mapping 6
S5 Filtering, QRS Subtraction and Sinusoidal recomposition 6
S6 Hilbert Transform Phase Reconstruction and PS Detection Methods 9
S7 Phase singularity and wavelet detection and tracking 11
S8 Renewal Process Models 14
S9 Testing the temporal stability (stationarity) of PS and wavelet rates of formation and destruction 14
Stationarity analysis 15
Random ensemble creation 18
S10 Introduction to Markov birth-death processes 19
Markov chains 19
Markov birth-death processes 20
The M/M/∞ Markov birth-death transition matrix 21
The eigenvalue spectrum, spectral gap and mixing rates of a Markov birth-death process 22
S11 Coarse graining approach to investigate effect of spatial density 24
Part 2: Supplementary results 25
S12 χ^2 Goodness of Fit Test for Computed Population Distribution of PS and Wavelets 26
S13 Correlation of PS and wavelet rate constants of formation and destruction 30
S14 Effect of PS/Wavelet Detection and Tracking Parameters on Measured Population Dynamics 31
Number of tracked frames τ 31
Size of tracking radius r 31
S15 Origins and stationarity of λf and λd 34
Sample Autocorrelation Function - All Cases 37
Coefficient of variation of λf ***and*** λd 49
S16 Validation using a secondary PS detection algorithm 51
S17 Stationarity and Population Dynamics for Long Lasting PS and Wavelets 53

PART 1: SUPPLEMENTARY METHODS

S1 Computer Simulations of Atrial Fibrillation

The Courtemanche model of AF used here was adapted from one previously described.[1] The Courtemanche model of the human atrial cardiomyocyte was used,[2] that was adapted and implemented in as a monodomain model in CARP, running in a parallel cloud-based cluster. Tissue conductance was set to provide physiological anisotropy with conduction velocity in a longitudinal direction of 47.9 cm/s. The tissue slab was 7x6cm in size, with longitudinal fiber anisotropy with grid ration of 6. Grid discretization was performed at 100μm resolution. The membrane capacitance was set to 1μF/cm.[2] Differential equations were solved with a 25μs timestep, with simulations of up to 5 seconds. Atrial fibrillation epochs were initiated by a standard S1-S2 cross-shock protocol. 2D simulations were performed with a ACh distribution was generated by randomly assigning a value between 0 and 0.001 μM to each node (cardiomyocyte). The ACh-activated K+ current was implemented using the previously-published model by Kneller et al.[3] AF epoch transmembrane voltage movies were visualized in Meshalyzer (OpenCARP), and exported as transmembrane voltage movies.

S2 Patient Baseline Characteristics- AF Cohort

Age (years)	62±8
Male, n (%)	21/26 (81%)
BMI (kg/m^2)	28.7±4.6
CHA_2DS_2VaSC	1.2 (1.2)
Persistent AF, n (%)	18 (69%)
Paroxysmal AF, n (%)	8 (31%)
LVEF (%)	53±11
LA diameter (mm)	45±8

Supplemental Table 1 showing baseline characteristics of patients enrolled from Flinders Medical Centre and University Medical Centre Hamburg.

S3 Sheep Atrial Fibrillation Model

The sheep atrial fibrillation model has previously been described.[4, 5] Right atrial tachypacing (16 weeks, (≥300 beats per minute) was used to obtain persistent AF. An electrophysiology study was undertaken under general anaesthesia using a 64-electrode constellation catheter (48 mm). Bipolar electrograms were filtered from 30 Hz to 500 Hz, and sampled at 1000 Hz. For each sheep, ≥20 min of intracardiac data were obtained in the left and right atrium.

S4 Rat AF – Optical mapping

The rat model of AF used in this study has previously been described.[6] Male Wistar rats weighing 200-275 g were injected subcutaneously (SC) with preoperative buprenorphine (0.03 mg/kg) and anaesthetized with 2% isoflurane. Under endotracheal intubation and assisted ventilation, a left thoracotomy was performed, followed by ligation of the left anterior descending coronary artery with 6-0 silk. The thorax was sutured using a 3-0 silk and the skin was stapled using metal clips. Buprenorphine (0.03 mg/kg) was injected SC 6 and 12 hours postoperatively. The same day, rats underwent transesophageal electrophysiological study (EPS). Langendorff perfused hearts underwent electrophysiology study and optical mapping. The heart was excised and perfused retrograde via the aorta with Krebs solution at 10 ml/min and 37C. After a 20-minute stabilization period, a recirculating solution containing 15 mmol/l blebbistatin was perfused for the purpose of mechanical contraction suppression. Di-4-ANEPPS (10 mmol/l, 0.1 ml) was introduced. Fluorescence signals were recorded at 2 kHz with a charge-coupled device focused on a region up to 8 x 8 mm in the RA or LA free-wall. AF-inductions were performed at BCLs 300, 250, 150, 100, 80, and 60 ms (3 s each) and with 25-Hz burst-pacing.

S5 Filtering, QRS Subtraction and Sinusoidal recomposition

Baseline drift was removed from signals by removing the best straight-fit line (detrending). A template subtraction method was used to remove far field ventricular depolarisation as described previously[7], following baseline correction of each epoch. Specifically, the template subtraction method identifies fiducial points for ventricular

complexes using the QRS detection algorithm by Pan and Tompkins[8]. An average or 'median' ventricular complex was then constructed by aligning the detected ventricular complexes at their respective fiducial points, and performing a median operation of the matching points in all complexes [7]. Subtracting the median complex from each ventricular complex resulted in QRS subtracted electrograms (Supplemental Figure 1). Further pre-processing was applied using a 3rd order Butterworth fitted with a 40-250 Hz band pass filter, and a 8th order Butterworth filter fitted with 10 Hz low pass filter applied in forward and reverse mode.[5, 9]

Following this, sinusoidal recomposition of the signal was applied to transform the AF electrogram signal into a sum of sinusoidal wavelets (Supplemental Figure 1). Doing so increases the accuracy of the phase reconstruction using the Hilbert transform, as mathematically this transformation works best on signals with a sinusoidal morphology (e.g. ventricular signals). As atrial signals are characterised by long iso-electric intervals between consecutive deflections, these can result in artifacts in the reconstructed phase. The sinusoidal recomposition transforms AF electrograms into sinusoidal wavelets which only exist when there is a negative derivative in the original signal (as a negative slope in a unipolar electrogram corresponds to the passing of a wavefront). The amplitude of the wavelet is proportional to the slope of the signal, and has a period equal to the mean cycle length of the electrogram (which was derived from the dominant frequency of the electrogram). These properties result in more accurate phase reconstruction for subsequent PS analyses.

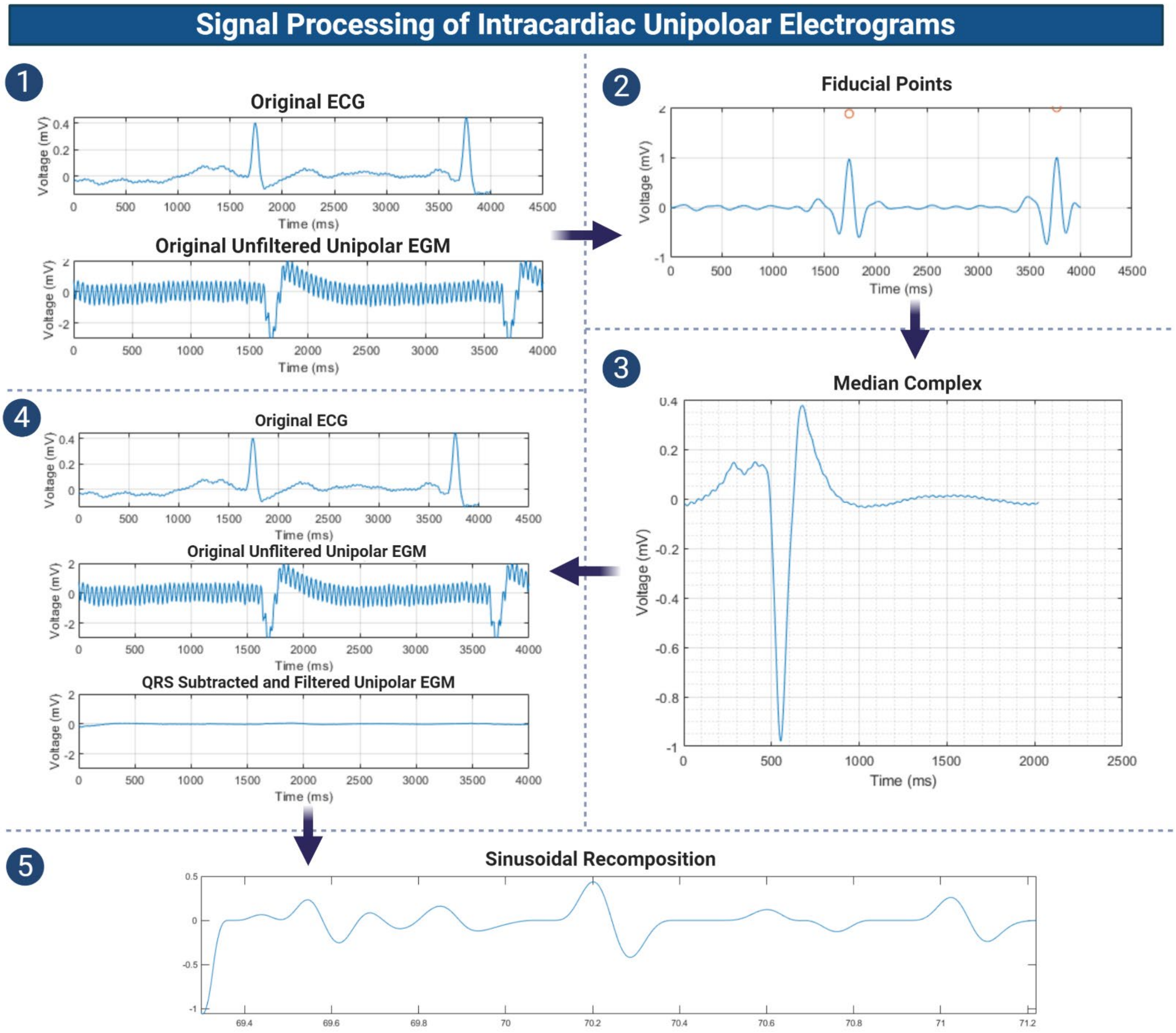

Supplemental Figure 1

S6 Hilbert Transform Phase Reconstruction and PS Detection Methods

In the context of cardiology, phase can be used to study electrical wave propagation. In this study, the instantaneous phase for each electrogram was reconstructed by applying the Hilbert transform on the cleaned and sinusoidally reconstructed signal for intracardiac unipolar EGM[10, 11], and using the transmembrane voltage for optically mapped and computer simulated AF. Mathematically, the Hilbert transform can be given as follows:

$$\phi(t) = arctan\left(\frac{-u(t) - u*}{H(u)(t) - u*}\right)$$

where $u*$ sets the origin of the phase plane with respect to the phase that is computed. [11] After applying the Hilbert transform, the instantaneous phase was interpolated using complex vector interpolation to avoid incorrect phase calculation. [9, 12]

Plotting the phase values at each recorded site of the heart for all time points t results in phase maps such as those shown in Supplemental Figure 2. Phase is colour coded, allowing visual interpretation of the propagation of electrical activity. An example of a single, localised rotor is shown in Supplemental Figure 2A, whereby the beginning of an action potential cycle corresponds to a phase of –π shown in blue, and the end of the cycle with a phase +π shown in red. The rotational movement of the rotor can clearly be seen. Similarly, Supplemental Figure 2B shows phase maps for human persistent AF. Propagation in this instance shows multiple complex wavefronts.

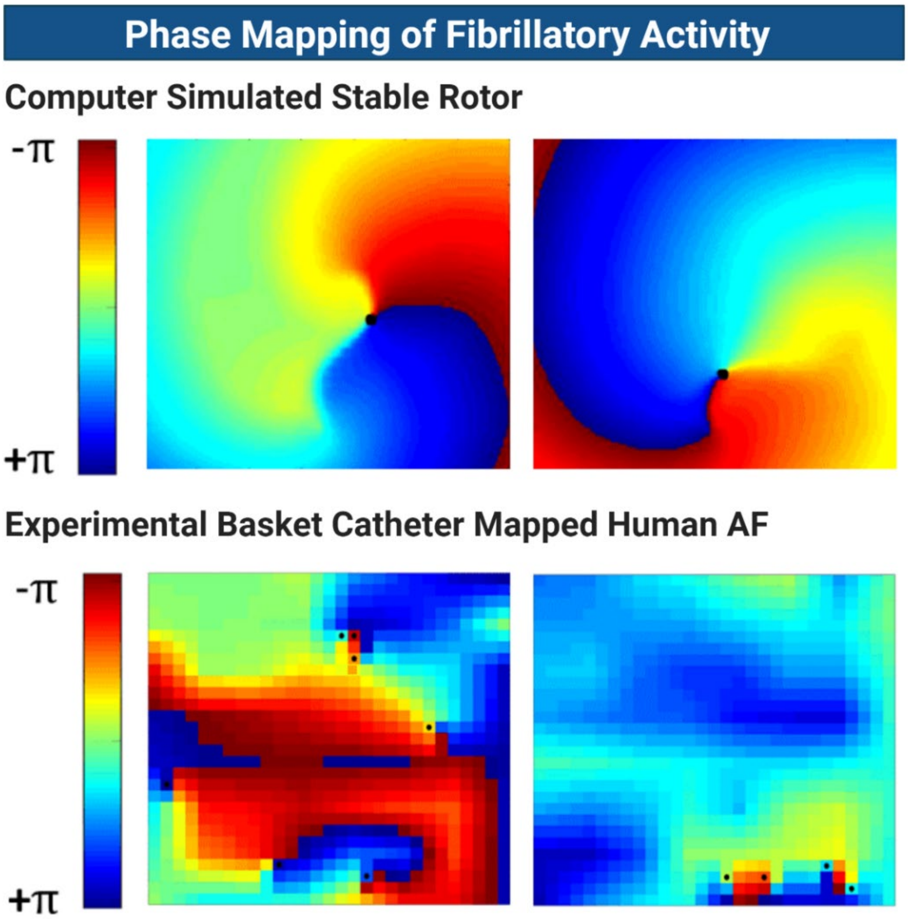

Supplemental Figure 2

S7 Phase singularity and wavelet detection and tracking

In this study, we use two approaches to detect PS: (i) a convolution kernel method[10, 13] and (ii) an extended topological charge method[14]. The convolution kernel method approximates the gradient of phase from the discretized phase map using a finite difference operation in the x and y directions, given by:

$$k_x[m,n] = \nabla_{\phi_x}[m,n] = \phi[m+1,\ n] - \phi[m,\ n]$$

$$k_y[m,n] = \nabla_{\phi_y}[m,n] = \phi[m,\ n+1] - \phi[m,\ n]$$

where is ϕ phase, and $[m,n]$ the pixel coordinates. The line integral at the pixel $[m,n]$ can there be approximated using the convolution kernel equation:

$$(\nabla X\vec{k}) \cdot \hat{z} \propto \nabla_x \otimes k_y + \nabla_y \otimes k_x$$

where $\otimes$ is the convolution operator, and ∇_x and ∇_y and convolutional kernels.[13, 15]

For cross validation, the second extended topological charge algorithm was used, which is also based on calculating the line integral of the phase gradient:

$$\oint_c \nabla \phi d\vec{l}$$

where ∇ is the spatial derivative, c is a closed loop surrounding a given pixel and ϕ is phase. The closed loop c comprises of an inner 2x2 path, surrounded by a 4x4 outer path. In a small 2 x 2 ring, PS detection translates to the presence of just a single phase difference greater than π, which is half of the full 2π cycle. The PS is said to exist at the intersection of the four pixels constituting the inner 2 x 2 ring. As using a single 2 x 2 ring may encompass more than one wave and cause artificial phase transitions, the second ring is added to ensure a PS is only identified if it is

simultaneously detected by both the inner and outer ring.[14] This results in greater noise insensitivity. Results are provided in the ‘Supplementary Results’ section (*Validation using a secondary PS detection algorithm,* page 51). To detect wavelets, we looked at wavefronts which were identified as lines of zero phase, and then calculated the wavelet lengths via summation of the distance between pixels located along the wavefront.

In order to track PS, a tracking algorithm was implemented as previously described[5]. New PS were defined as the detection of a PS not falling within the surrounding radius of r of an existing PS for a duration of τ. As default, τ was set to 10ms (20 frames) and r set to 80 in computer simulated AF, 4 in basket AF, 2 in HD-grid AF and 20 in optically mapped AF to account for the varying spatial resolution of the mapped field.

For wavelets, a graph theory approach was implemented as described by Rogers[16], which allowed identification of new wavelets, as well as wavelet splitting and merging events. In short, the algorithm tracks the wavelets present throughout each time-step of the recording and records their anchor points (start and end (x,y) coordinates). The anchor points of wavelets in the current time-step are compared to the anchor points of every wavelet in the next time step and then will compare to find any matches. If a match has occurred, then the wavelet is said to be the same and if not, it is tagged as new.

To determine wavelet and PS lifetime and inter-formation times, a look-up table indexing onset time, offset time and electrode location for each PS and wavelet was created[5]. If the PS or wavelet does not fall within a radius r of another, the PS/wavelet is given a new ID by incrementing the list of current PS and wavelet IDs by 1. The

look-up table will then record the current time stamp as the first time the PS or wavelet is detected, and records all consequent time stamps it remains present. The look-up table also records the x and y locations of the PS and wavelet on the phase map. For PS, this is given by a paired (x,y) coordinate value, while for wavelets the anchor points (x and y coordinates of points at the tail ends of the wavelet) are recorded, as well as the (x,y) coordinate of the middle of the wavelet (mid-point). Using this look-up table information, the total time the PS or wavelet is present (lifetime) can be measured, as well as the time taken between the creation of the current PS/wavelet until the creation of the next consecutive PS/wavelet (inter-formation time).

To cross-validate the effect of PS tracking and detection parameters on PS and wavelet population dynamics, various values of τ and r were tested. Results are provided in the 'Supplementary Results' section (*Effect of PS/Wavelet Detection and Tracking Parameters on Measured Population Dynamics*, page 48).

S8 Renewal Process Models

We model PS and wavelet destruction and formation as renewal processes.[5] For PS and wavelet destruction, we measured the waiting times for an existing PS to be destroyed, and for PS and wavelet formation, we studied the waiting times between the creation of new phase singularities. PS lifetime and inter-formation event times are random variables generated according to an exponential distribution, with rate parameter λ. The probability density function for all PS and wavelet lifetimes or inter-formation times is given by:

$$f(t) = \{\lambda e^{-\lambda t} \quad t \geq 0\}$$

where t is time, and λ the PS destruction or formation rate (referred to as λ_f and λ_d respectively).

S9 Testing the temporal stability (stationarity) of PS and wavelet rates of formation and destruction

Atrial fibrillation, particularly in its clinically significant persistent forms, is characterized by turbulent dynamics of rotors and wavelets representing a form of underlying chaos occurring in an anatomically closed system.[17] However, we reasoned that over time, the formation and destruction of new PS and wavelets should converge to stable rates given the uncorrelated electrical wave propagation in AF, and this is our hypothesis. In a statistical sense, a long-term average rate arises if the timing between events is random or statistically independent (a property known as '*memorylessness*').[18] This leads to a constant instantaneous probability, and a stationary or stable long-term average rate[18].

To investigate this hypothesis, stationarity of the PS and wavelet inter-event series was analyzed to determine statistical independence of new PS or wavelet

formation and destruction times. Specifically, autocorrelation was assessed using the sample autocorrelation function of the univariate inter-event series of PS and wavelet lifetimes and inter-formation times, given by measuring the correlation between the event series y_k and $y_{k+\tau}$ (a shifted or 'lagged' version of itself) (Supplementary Materials).

As an additional test for stationarity, 1000 random ensembles were constructed by indexing a random PS or wavelet as the starting index of the inter-event series. If constant rates λ_f and λ_d arise due to the memoryless property, then λ_f and λ_d calculated across the random ensembles should converge to a constant rate (Supplemental Data).

To further validate the stability of λ_f and $\lambda_{d,}$ the coefficient of variation (CV) of λ_f and λ_d was calculated as the ratio of the standard deviation to the mean (SD/mean). For comparison with established clinical measures, CV for dominant frequency (DF) and the AF cycle length (AFCL) was also calculated. Dominant frequency (DF) analysis was performed as described [19]. The regularity index (RI) at each DF was obtained, and only DF possessing an RI>0.2 were included in analyses[19]. AFCL was annotated by two trained electrophysiologists.

Stationarity analysis

Stationarity of the PS and wavelet inter-event series was analyzed to determine statistical independence of new PS or wavelet formation and destruction times. Stationarity is evident if the mean function $\mu_x(k)$ is not a function of PS/wavelet event number, k, and thus can be given by [20]:

$$\mu_x(k) = \mu_x, for\ all\ k\ \in \mathbb{R}$$

In addition, the autocorrelation function should only depend on difference $\tau = k_1 - k_2$ (and thus be independent of individual events k_1 and k_2) such that:

$$R_X(k_1, k_2) = R_X(k_1 - k_2) = R_X(\tau), for\ all\ k_1, k_2 \in \mathbb{R}$$

where R_X is the autocorrelation function of $\{X(k)\}$, k the event number and τ the lag[20].

Specifically, autocorrelation was assessed using the sample autocorrelation function of the univariate inter-event series of PS and wavelet lifetimes and inter-formation times, given by measuring the correlation between the event series y_k and $y_{k+\tau}$ (a shifted or 'lagged' version of itself) (Supplemental Figure 3). In essence, this would mean the correlation between the lifetime or inter-formation time of PS_k (or $Wavelet_k$) is assessed against the lifetime or inter-formation time of $PS_{k+\tau}$ (or $Wavelet_{k+\tau}$). If the ordered series of PS and wavelet inter-event times show autocorrelation approaching zero for all non-zero lags, this suggests that the lifetimes/inter-formation times between events is random or statistically independent (a property known as '*memorylessness*'). This leads to a constant instantaneous probability, and a stationary or stable long-term average rate of PS/wavelet formation and destruction.[18]

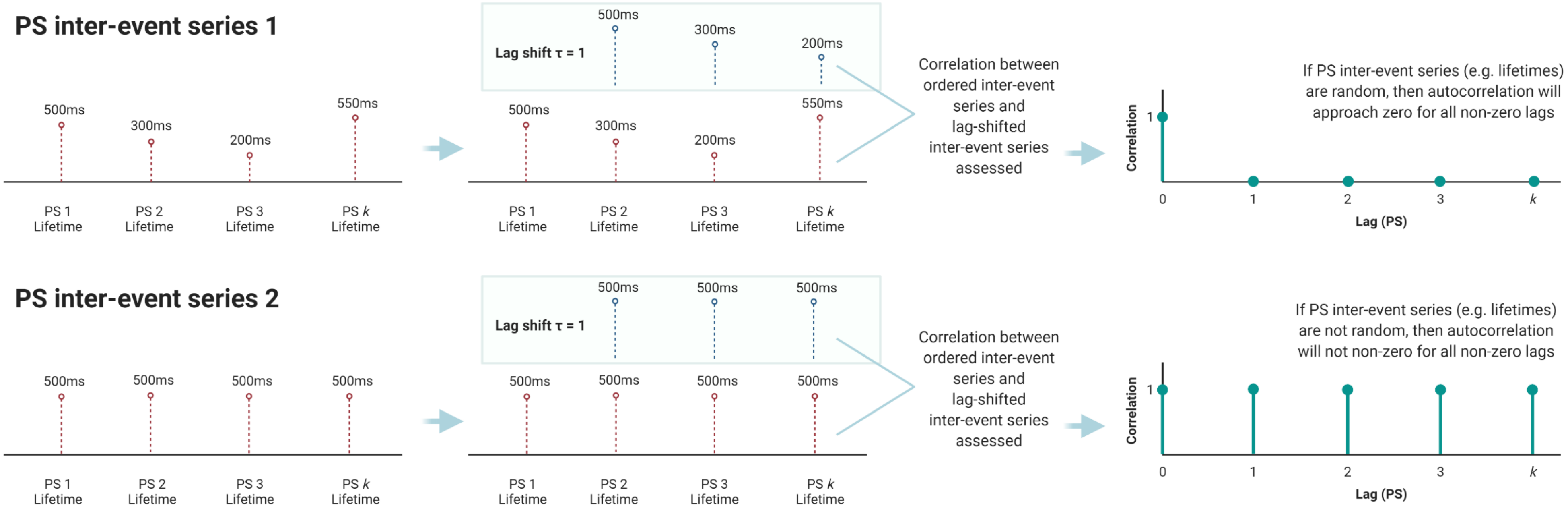

Supplemental Figure 3

Random ensemble creation

λ_f and λ_d were estimated for randomly constructed short-duration windows, and compared to the long-term average λ_f and λ_d. To create random windows of size n, a random PS was selected as the starting index. For each window size, 1000 random ensembles were created, and PS lifetimes and inter-formation times calculated. A schematic outlining the creation of random windows is shown in Supplemental Figure 4.

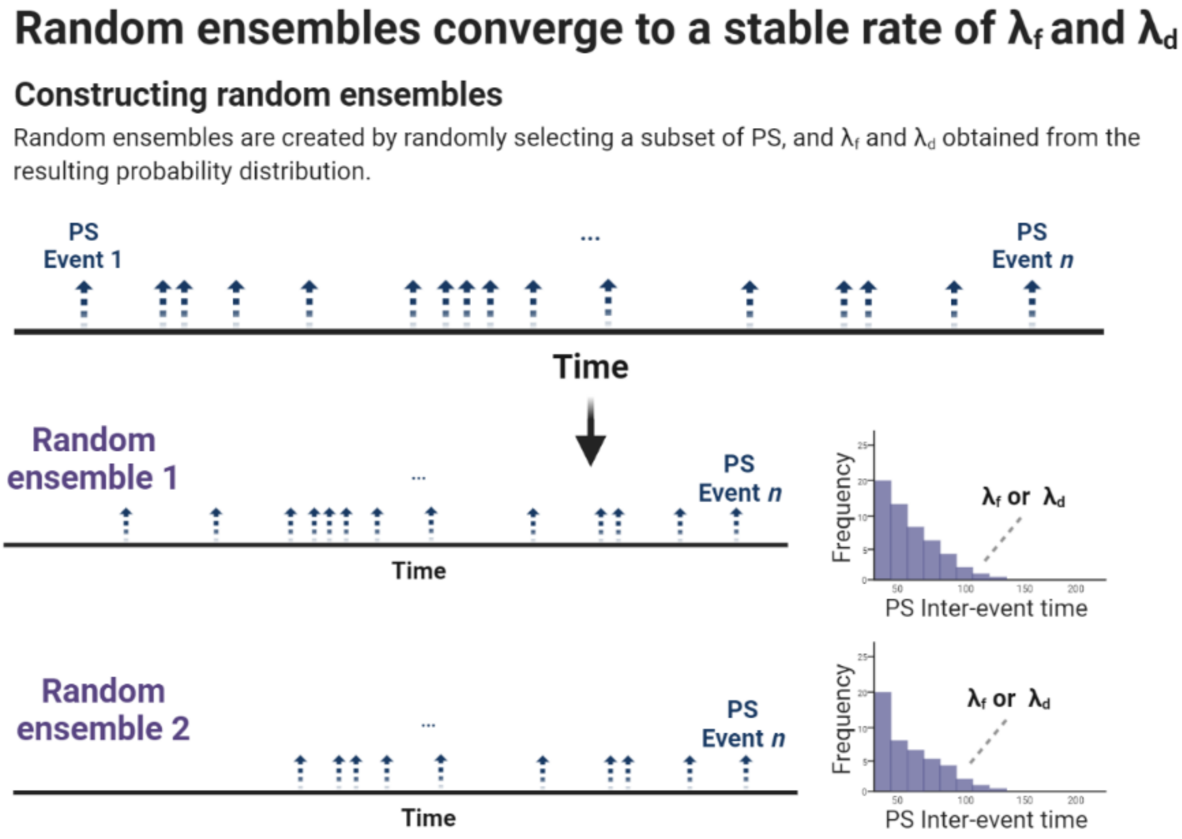

Supplemental Figure 4

S10 Introduction to Markov birth-death processes

Markov chains

Markov chains are mathematical systems used to model stochastic (random) processes. Specifically, a Markov chain models transitions from one state to another according to probabilistic rules. These states may be a set of values or a situation.[18] The process begins at one of these states and moves from one state to another, with each move referred to as a '*step*'. If the chain is currently at state s_i, it will move to state s_j with a probability $p_{i,j}$. This probability is referred to as a 't*ransition probability'*, and will only depend on the current state and not any of the previously occupied states (this is known as the '*Markovian*' or '*memoryless*' property). The process can also remain in its current state, which occurs with a probability $p_{i,i}$.

The probability distribution of these state transitions can be given by the Markov chain's '*transition matrix*'. The transition matrix will be $N \text{ x } N$ in dimension, whereby N is equal to all possible states. The rows of this matrix must also sum to 1, as each row represents a unique probability distribution.

To define an example Markov chain, let's consider the weather in Adelaide. For simplicity, the weather can assume only one of two states: sunshine or rain. If we assume that tomorrow's weather will be the same as today, and this assumption is correct 75% of the time (irrespective of whether the weather today is sunshine or rain), then the Markov chain state transition diagram can be given by Supplemental Figure 5.

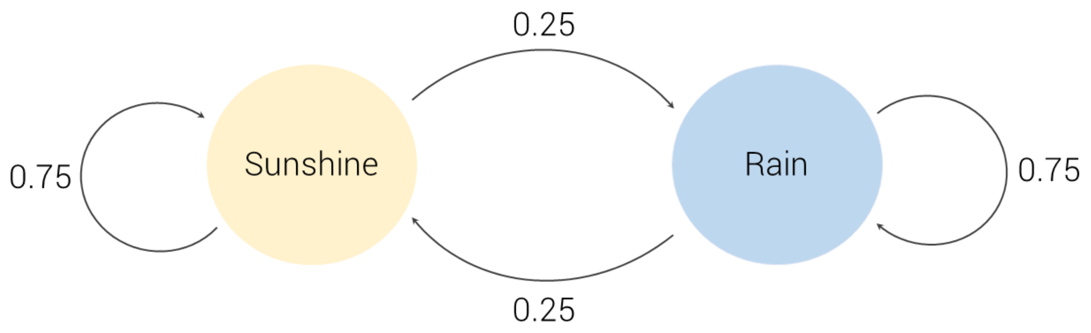

Supplemental Figure 5: Markov chain state transition diagram

The corresponding transition matrix can thus be given by:

$$\begin{bmatrix} 0.75 & 0.25 \\ 0.25 & 0.75 \end{bmatrix}$$

Markov birth-death processes

A birth-death process is a Markov chain used to represent the population of particles in a system[18], and are commonly used to study various aspects of population growth in biology[21]. In this study, we used Markov birth-death processes to model the birth and death of phase singularities (PS) in human, experimental and computer simulated AF. We hypothesized that given that PS renewal rate constants are stationary (S8 and S11), an M/M/∞ birth-death process could account for the PS number and duration in AF recordings.

In an M/M/∞ process, the first 'M' denotes a memoryless (stationary) renewal processes of PS formation (λ_f), the second 'M' denotes a memoryless renewal process of PS destruction (λ_d), and the ∞ denotes the concept that arrivals into the system are immediately available for service. In the case of AF phase singularities, this means as soon as they are formed, they become immediately available for potential annihilation.

The M/M/∞ birth-death process has mathematically derived steady-state equations for average population number, and population distribution given by equations 1 and 2 respectively:

$$N = \frac{\lambda_f}{\lambda_d} \qquad (1)$$

$$P_n = \frac{\left(\lambda_f/\lambda_d\right)^n e^{-\lambda_f/\lambda_d}}{n!} \qquad (2)$$

From equation 1, we can see that the number of PS and wavelets in a system is governed by the interplay between the rates λ_f and λ_d. If the rate of formation is exactly equal to the rate of destruction, PS and wavelets will be destroyed as soon as they are formed, meaning none would be observed. It is thus the ratio between the formation rate and destruction rate that governs how many entities are in the system, which should be >1 for AF to sustain.

The M/M/∞ Markov birth-death transition matrix

The following section is a brief illustration of the M/M/∞ birth-death transition matrix adapted from Crawford, 2018.[22] As mentioned previously, birth-death processes count the population in a system at a time t. For an M/M/∞ birth-death process, we consider the behavior of the system by considering a short time dt when there are k particles (in our case PS, or wavelets in the system). As *dt* approaches zero, the probability of an event in the interval $(t, t + dt)$, given $X(t) = k$, and $\mu_k = k\mu_1$:

$$P_{ab}(t + dt) = \lambda_{b-1}P_{a,b-1}(t)dt + \mu_{b+1}P_{a,b+1}(t)dt + (1 - \lambda_b - \mu_b)P_{ab}(t)dt + o(dt)$$

In matrix form the Markov transition or probability matrix is therefore:

$$P = \begin{bmatrix} P_{i,j} & P_{i,j+1} \\ P_{i+1,j} & P_{i+1,j+1} \end{bmatrix}, \quad P_{i,j} = \begin{cases} p_i, & if\ j = i+1 \\ q_i, & if\ j = i-1 \\ 1 - p_i - q, & if\ j = 1 \\ 0, & else \end{cases}$$

$$P = \begin{bmatrix} P_{0,0} & P_{0,1} & P_{0,2} & P_{0,3} & P_{0,4} & P_{0,5} & P_{0,6} \\ P_{1,0} & P_{1,1} & P_{1,2} & P_{1,3} & P_{1,4} & P_{1,5} & P_{1,6} \\ P_{2,0} & P_{2,1} & P_{2,2} & P_{2,3} & P_{2,4} & P_{2,5} & P_{2,6} \\ P_{3,0} & P_{3,1} & P_{3,2} & P_{3,3} & P_{3,4} & P_{3,5} & P_{3,6} \\ P_{4,0} & P_{4,1} & P_{4,2} & P_{4,3} & P_{4,4} & P_{4,5} & P_{4,6} \\ P_{5,0} & P_{5,1} & P_{5,2} & P_{5,3} & P_{5,4} & P_{5,5} & P_{5,6} \\ P_{6,0} & P_{6,1} & P_{6,2} & P_{6,3} & P_{6,4} & P_{6,5} & P_{6,6} \end{bmatrix}$$

$$P = \begin{bmatrix} 1 - p_i - q_i & p_i & 0 & 0 & 0 & 0 & 0 \\ q_i & 1 - p_i - q_i & p_i & 0 & 0 & 0 & 0 \\ 0 & q_i & 1 - p_i - q_i & p_i & 0 & 0 & 0 \\ 0 & 0 & q_i & 1 - p_i - q_i & p_i & 0 & 0 \\ 0 & 0 & 0 & q_i & 1 - p_i - q_i & p_i & 0 \\ 0 & 0 & 0 & 0 & q_i & 1 - p_i - q_i & p_i \\ 0 & 0 & 0 & 0 & 0 & q_i & 1 - p_i - q_i \end{bmatrix}$$

$$For\ an\ M/M/\infty\ queue\ where\ number\ of\ servers\ k > 1, \quad p_i = \lambda, \ q_i = \begin{cases} i\mu, & if\ i < k \\ k\mu, & if\ i \geq k \end{cases}$$

$$P = \begin{bmatrix} 1 - \lambda_f & \lambda_f & 0 & 0 & 0 & 0 & 0 \\ \lambda_d & 1 - \lambda_f - \lambda_d & \lambda_f & 0 & 0 & 0 & 0 \\ 0 & 2\lambda_d & 1 - \lambda_f - 2\mu & \lambda_f & 0 & 0 & 0 \\ 0 & 0 & 3\lambda_d & 1 - \lambda_f - 3\lambda_d & \lambda_f & 0 & 0 \\ 0 & 0 & 0 & 4\lambda_d & 1 - \lambda_f - 4\lambda_d & \lambda_f & 0 \\ 0 & 0 & 0 & 0 & 5\lambda_d & 1 - \lambda_f - 5\lambda_d & \lambda_f \\ 0 & 0 & 0 & 0 & 0 & 6\lambda_d & 1 - \lambda_f - 6\lambda_d \ldots \end{bmatrix}$$

The eigenvalue spectrum, spectral gap and mixing rates of a Markov birth-death process

The transition matrix of a Markov birth-death process is often of interest as it can be used to study the long-term population distribution of a system. This is referred to as the '*steady state distribution*', which approaches a stable limiting distribution (i.e. equilibrium) when observed over long periods of time. Mathematically speaking, this steady state distribution can be identified by finding the eigenvector which has at least one eigenvalue (λ) = 1. In simple terms, an eigenvector is a vector that does not

change in direction when a linear transformation is applied to it, whilst eigenvalues are the scalar quantities that define how the eigenvector is scaled due to the given linear transformation.

Studying the eigenvalues for a given transition matrix, i.e. the *'eigenvalue spectrum'*, can give insights into the dynamics of that system. For example, the difference between the first and second largest eigenvalues is referred to as the '*spectral gap*' and describes how quickly the steady state distribution is reached. The spectral gap is given by[23]:

$$1 - z$$

where 1 is the largest eigenvalue and z the second largest eigenvalue modulus (often referred to as SLEM).

The time taken to reach the steady state distribution can also be expressed in terms of the '*mixing rate*' of the birth-death process, given by[23]:

$$\log(1 - z)$$

If SLEM is very close to 1, then the mixing rate will approximate the spectral gap.

In this study, we specifically hypothesized that AF termination would occur due to a deviation from the steady state of AF dynamics, due to the steady state distribution being reached more slowly. This consequently would provide a greater opportunity for the process to diverge from the steady state distribution and break the cycle of PS and wavelet regeneration.

S11 Coarse graining approach to investigate effect of spatial density

In general terms, 'decimation' or 'down sampling' is a process in which the sampling rate is reduced. In the context of AF mapping, this refers to reducing the number of pixels sampled to produce the resultant voltage and phase maps. As clinical settings often make use of mapping modalities that cannot sample every point of the atria and hence have limited spatial density, we wanted to investigate whether spatial density would affect the PS and wavelet formation and destruction rates measured, as well as the population dynamics observed.

To study this, spatially uniform pixels were selected at progressively coarse-grained spatial densities (i.e. sampled at lower and lower densities) in computer simulated AF. Following this, the simulated membrane voltage between the remaining pixels was interpolated to produce voltage maps. Instantaneous phase was calculated as previously described in S5, and PS/wavelet detected as described in S6. To look at the effect of spatial density on PS and wavelet formation/destruction, λ_f and λ_d were measured as described in S7 and PS and wavelet population dynamics studied using the steady state equations as described in S9.

PART 2: SUPPLEMENTARY RESULTS

S12 χ^2 Goodness of Fit Test for Computed Population Distribution of PS and Wavelets

Computed PS and wavelet population distributions were fitted to the observed population distribution using squared (χ2) goodness of fit testing. Computed population distributions were calculated using the M/M/∞ steady state equation given by:

$$P_n = \frac{\left(\lambda_f/\lambda_d\right)^n e^{-\lambda_f/\lambda_d}}{n!}$$

where n re phase singularity or wavelet population size, and λ_f and λ_d the rate of PS/wavelet formation and destruction respectively. The observed population distribution of PS and wavelets was given by the amount of time n number of PS or wavelets was present throughout the recorded epoch.

Data fitting was performed in Matlab, using the probability density function (PDF) of each case. χ2 goodness of fit test was used to assess the adequacy of fit, which uses the null hypothesis that the data evaluated comes from an exponential distribution. The degrees of freedom were set to number of categories - 2 (for a Poisson distribution), and the significance level to reject the null hypothesis α = 0.05.

In all cases and all model systems, computed population distributions fit well to the observed population distribution (P > 0.05 in all cases). Goodness of fit test statistics for all human basket catheter-mapped cases are provided in Supplemental Table 2.

Supplemental Table 2: χ2 goodness of fit test statistics for basket-mapped human AF

Basket Human AF					
PS χ2	PS df	PS p-val	W χ2	W df	W p-val
13.23037874	7	0.0666903	14.8549361	6	0.021415
14.53416954	7	0.0424565	15.90749603	6	0.014259
0.962103613	6	0.9870082	1.03008797	5	0.960103
10.68751949	7	0.152842	17.600095	6	0.007313
4.709815748	8	0.7880932	12.97167636	9	0.163892
6.241509049	8	0.6201993	11.4851244	8	0.175697
0.781739652	5	0.9781834	1.673386499	6	0.947157
7.808658839	9	0.553538	20.35089354	8	0.009087
1.002674519	6	0.9855107	1.250496604	6	0.974317
3.628721614	8	0.8889732	3.990393789	6	0.677976
2.924567728	7	0.8918915	4.891318245	5	0.429287
2.774276619	7	0.905069	10.72714105	6	0.097184
3.049316349	8	0.9312246	13.94285524	6	0.030281
3.674931975	9	0.9314769	14.22745203	7	0.047281
10.97855663	8	0.2029172	21.19640593	8	0.006644
6.160806008	8	0.6292246	7.503205318	8	0.483436
8.153000864	9	0.5188041	9.334321751	8	0.314883
7.502608017	8	0.4834979	8.404577054	7	0.298273
7.594752467	8	0.4740218	8.233076859	8	0.41104
8.556855129	10	0.5746108	10.35862355	9	0.32224
8.370346471	9	0.4972975	9.224758244	8	0.323692
8.296525744	8	0.4050537	8.629456215	7	0.280365
4.967529937	6	0.5479844	7.62526174	5	0.178133
7.889849226	7	0.3424071	7.467741621	7	0.381853
4.781312174	7	0.6866305	9.225108027	6	0.161308
7.811249987	9	0.5532741	11.0706885	5	0.049996
10.19854559	8	0.2513663	11.94307109	7	0.102447
12.56281626	7	0.0835043	16.53944415	6	0.011134
8.147943181	15	0.9177145	7.454410493	7	0.383146
10.05336626	7	0.1855694	15.63554077	5	0.007965
7.103444457	8	0.5255141	9.597492854	7	0.212553
4.165496169	7	0.7605304	7.010864712	6	0.319844
4.472519729	7	0.7240238	6.321364335	5	0.276192
4.546407825	7	0.715122	5.227549376	7	0.632216
3.248245318	8	0.9178268	5.726900107	7	0.571975
6.201492148	8	0.6246731	17.29858946	6	0.008246
7.829107291	7	0.3479041	16.09842131	5	0.006569
8.560610822	7	0.2857594	16.52920497	6	0.011178
2.961216285	7	0.8885667	5.291586363	5	0.38134
2.984673206	6	0.810768	5.494289279	5	0.358572
2.314844176	6	0.8885869	5.2833327	4	0.259441
3.386220337	6	0.7590409	4.300110433	4	0.366911

2.648207055	5	0.7540278	3.686831796	4	0.450044
2.61728429	6	0.8551177	3.412277746	5	0.636701
2.08547241	7	0.9549584	4.38891029	6	0.624201
1.52685459	6	0.9576908	4.684991362	4	0.321172
2.951118357	8	0.9373875	3.887872901	7	0.792598
3.45801154	6	0.7495474	4.150546731	6	0.656312
3.912534691	8	0.8649274	5.226266655	7	0.632372
3.842093843	7	0.797775	4.563598256	7	0.713046
1.964768727	5	0.8539982	3.750158125	4	0.440873
3.414892876	7	0.844156	5.878487041	7	0.554007
2.615280161	6	0.8553495	5.375348872	6	0.496649
2.692745032	7	0.9118979	10.90731565	6	0.091284
3.60169282	8	0.8911556	7.206411808	8	0.514535
2.830055929	8	0.9445654	5.894106972	6	0.435156
2.809444295	6	0.8323554	5.15044558	4	0.272206
2.075881528	7	0.9555213	3.973057141	5	0.553302
3.079733854	7	0.8775295	3.995268203	5	0.550097
3.063087964	8	0.9303362	7.766537165	10	0.651629
3.133508389	8	0.9257017	4.838271984	8	0.774714
2.255500341	9	0.9867531	3.199485363	4	0.525014
2.842828396	8	0.9438302	4.236231273	7	0.752202
3.528834456	7	0.832162	4.163459447	7	0.760769
3.701766083	6	0.7169604	4.425102674	6	0.61935
2.928145386	6	0.8178098	3.35921536	5	0.644789

Supplemental Table 3: χ2 goodness of fit test statistics for HD-grid mapped human AF

HD-Grid Human AF					
PS χ2	PS df	PS p-val	W χ2	W df	W p-val
0.292090468	2	0.8641186	0.066663573	2	0.967218
0.464866867	2	0.7926025	0.06038831	2	0.970257
0.531307919	2	0.7667044	0.226626721	2	0.892871
0.3516776	2	0.8387532	0.125350959	2	0.971226
0.167477229	2	0.9196716	0.058391784	2	0.974232
0.37804357	2	0.8277685	0.052211964	2	0.800609
0.226612022	1	0.6340474	0.063785952	1	0.972331
0.091753393	2	0.9551597	0.056118938	2	0.891831
0.385494809	2	0.8246903	0.228956224	2	0.71791
0.848456981	2	0.6542744	0.100787848	2	0.164639
1.049236608	1	0.305683	16.18644266	1	0.202946
0.540459355	2	0.7632042	9.160948565	1	0.062657
0.388067944	2	0.8236299	5.456279585	1	0.064434
0.36949326	2	0.8313149	5.375098815	1	0.136174

0.540459355	2	0.7632042	0.267464347	3	0.966027
0.159939793	1	0.689212	0.223508277	1	0.63638
0.388067944	2	0.8236299	0.287215232	2	0.866228
0.521700178	2	0.7703964	0.970850827	2	0.615435
0.922803092	2	0.6303995	0.196362696	2	0.906484
1.382219543	3	0.7097078	1.0562162	2	0.58972
0.479037534	2	0.7870065	0.826487042	2	0.661501
0.302996343	2	0.8594195	4.477642341	3	0.214293
0.610376953	2	0.7369845	0.934398377	3	0.81712
0.36949326	2	0.8313149	0.834155606	2	0.65897
0.735333531	2	0.6923479	0.834936638	2	0.658712
0.522139748	2	0.7702271	0.144725305	2	0.930193
0.201415579	2	0.9041972	3.955300453	2	0.138394
0.20716972	1	0.6489949	0.188844765	1	0.66388
0.197579037	2	0.9059334	0.254951803	2	0.880315
0.479580446	3	0.923354	0.50853091	2	0.775486
0.152925829	1	0.6957549	1.049236608	1	0.305683
0.04538794	1	0.8312923	0.540459355	2	0.763204
0.288936929	2	0.8654822	0.159939793	1	0.689212
0.109509168	2	0.9467175	0.388067944	2	0.82363

Supplemental Table 4: χ2 goodness of fit test statistics for basket mapped sheep AF

Basket Sheep AF					
PS χ2	PS df	PS p-val	W χ2	W df	W p-val
15.4095481	8	0.616545	17.47699506	7	0.145663
19.70758762	11	0.4951582	24.72814294	9	0.328709
7.977470161	8	0.435674	38.92070798	7	0.23736
14.84695124	10	0.1377422	21.28882667	8	0.64185

Supplemental Table 5: χ2 goodness of fit test statistics for optically mapped rat AF

Optically Mapped Rat AF					
PS χ2	PS df	PS p-val	W χ2	W df	W p-val
0.277553784	6	0.9995984	0.314013591	4	0.988892
1.536478579	6	0.9570347	1.618160986	4	0.805524
9.754959973	12	0.637448	1.069718289	7	0.993624
9.361513005	12	0.6717815	1.026196321	7	0.994395
0.147804898	7	0.9999911	0.576545349	5	0.989052
0.145476898	7	0.9999916	0.561223073	5	0.98971

S13 Correlation of PS and wavelet rate constants of formation and destruction

To investigate the topological connection between PS and wavelet rates of formation and destruction, the relationship between λ_f and λ_d for PS and wavelets was investigated.

As shown in Supplemental Figure 6, λ_f and λ_d for wavelet and PS are highly correlated in keeping with the topological connection these two forms of propagation (λ_f: R^2 = ; P < 0.001; λ_d: R^2 = ; P < 0.001).

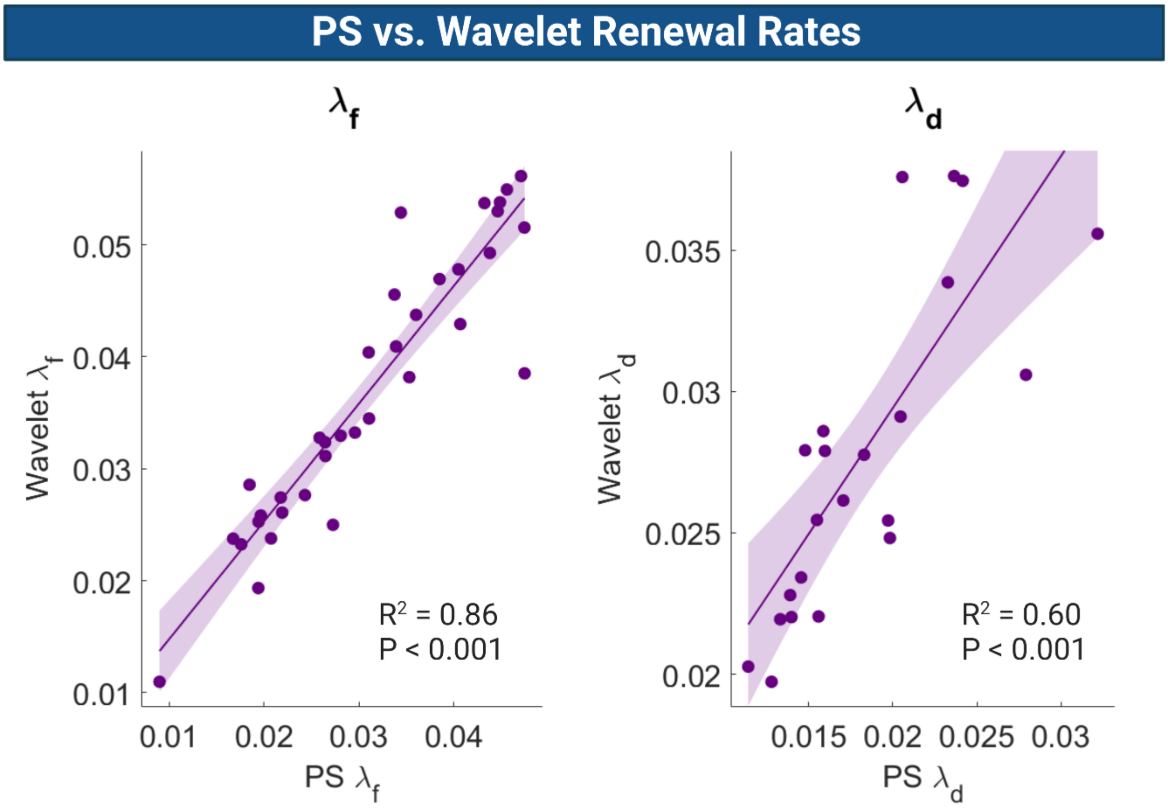

Supplemental Figure 6

S14 Effect of PS/Wavelet Detection and Tracking Parameters on Measured Population Dynamics

Number of tracked frames τ

For cross-validation, analyses were repeated in basket catheter-mapped human AF after varying the parameter τ of the PS partitioning algorithm such that a PS were required to be present for ≥ 10, 20, 40 and 80 consecutive frames (corresponding to durations of 5ms, 10ms, 20ms and 40ms respectively at a 2000Hz sampling frequency). As shown below, increasing the number of frames did not significantly alter computed λ_f and λ_d when compared to the default τ = 20, as λ_f and λ_d were strongly correlated in all cases (R^2 > 0.90). The PS number detected was also correlated for various values of τ (Supplemental Figure 7).

Size of tracking radius r

For extra cross-validation, analyses were repeated in basket catheter-mapped human AF after varying the parameter r of the PS partitioning algorithm such that a PS were tracked within the electrode neighbourhood of 2*sqrt(2), 4*sqrt(2), 6*sqrt(2) and 8*sqrt(2) pixels (corresponding to spatial distances of 2.83 pixels, 5.67 pixels, 8.49 pixels and 11.31 pixels on the interpolated 29x29 pixel grid). If a PS did not fall within the specified electrode neighbourhood of another PS within the duration τ, the PS would be tagged as a new PS and given a new ID. As shown below, increasing the tracking radius did not significantly alter computed λ_f and λ_d when compared to the default r = 4, as λ_f and λ_d were strongly correlated in all cases (R^2 > 0.90). The PS number detected was also correlated for various values of r, but becomes less correlates for much larger r (Supplemental Figure 8).

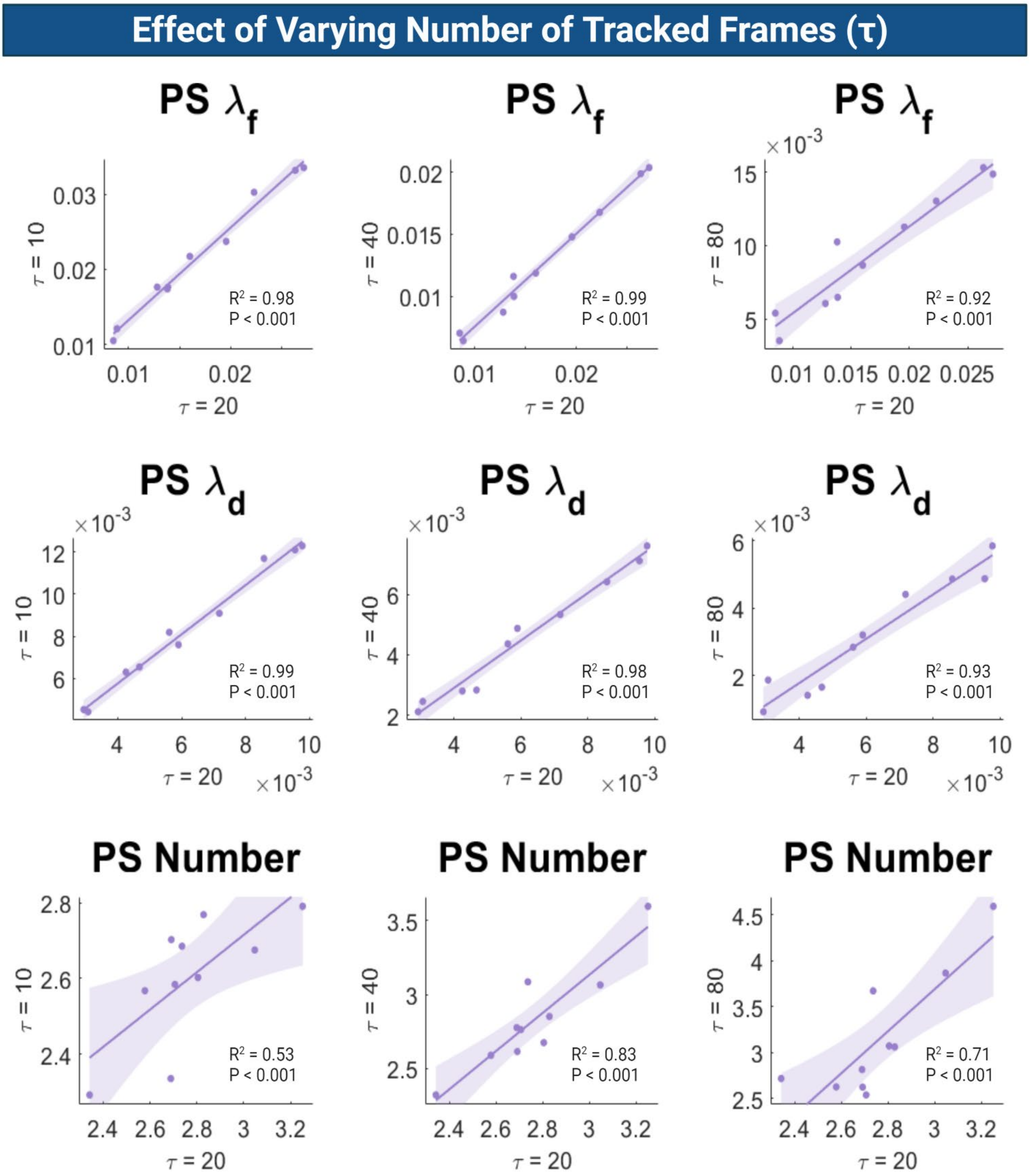

Supplemental Figure 7

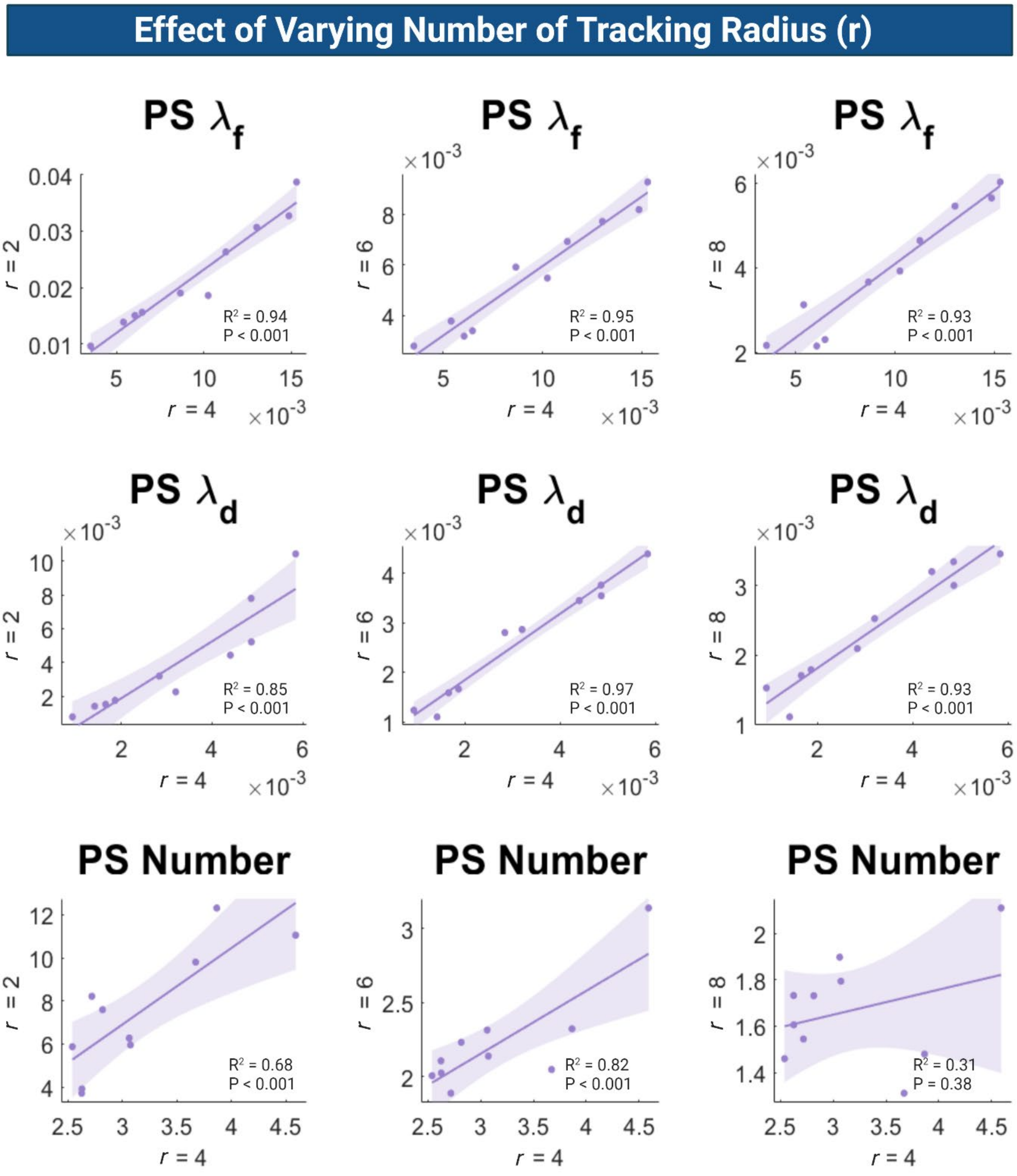

Supplemental Figure 8

S15 Origins and stationarity of λ_f and λ_d

To study the autocorrelation of PS formation and destruction, PS inter-formation times and lifetimes were considered as an ordered inter-event series, and correlograms constructed. All cases of human AF (mapped with basket catheter and HD-grid), optically mapped rat AF, sheep AF, and computer simulated AF demonstrated an autocorrelation close to zero for all non-zero lags (Supplemental Figure 9A-B), consistent with an underlying stable rate for λ_f and λ_d in all systems studied[20]. Correlograms for all human cases are shown in Supplemental Figure 10-Supplemental Figure 13.

λ_f and λ_d computed from short-duration random ensembles (<15 seconds) were correlated to those from full 5-minute epoch in human basket AF cases (Supplemental Figure 9C) with a coefficient of variation for λ_f of 3.63% (95%CI,1.5%,5.8%), which was significantly lower than the coefficient of variation for DF (16.57% (95%CI,3.0%,30%), P<0.001) and AFCL (12.3% (95%CI,6.7%,17.6%), P<0.001). The coefficient of variation for λ_d (2.5% (95%CI,1.5%,3.5%)) was also significantly lower than DF (P<0.001) and AFCL (P>0.001) (Supplementary Materials). When compared in all cases of human basket AF, λ_f and λ_d computed from random <15sec ensembles linearly correlated with λ_f (R^2=0.96; P<0.001) and λ_d (R^2=0.97; P<0.001) computed from the respective 5-minute recording with points lying along the line of identity, confirming stationarity (Supplemental Figure 9C).

Similar findings were also seen for λ_f and λ_d computed from the full epoch vs. short-duration random ensembles in computer simulated AF (λ_f: R^2=0.78, P<0.001; λ_d: R^2=0.90, P<0.001 full 1000 frame epoch vs. 100 frame window), sheep AF (λ_f: R^2=0.99, P<0.001; λ_d: R^2=0.99, P<0.001 full >5min epoch vs. <30sec window) and

optically mapped rat AF (λ_f: R^2=0.91, P<0.001; λ_d: R^2=0.99, P<0.001 full 1sec epoch vs. <500ms window).

Analyses were repeated with a secondary PS detection algorithm and various PS detection and tracking parameters (number of tracked frames τ and tracking radius r). Autocorrelation of PS inter-event series constructed using PS lifetimes and inter-formation times measured using the secondary PS detection algorithm also returned correlograms approaching zero for all non-zero lags, and λ_f and λ_d were linearly correlated when computed using various values of τ (R^2>0.90 for allτ, P<0.001) and r (R^2>0.80 for all r, P<0.001), implying consistency of measured rate constants (Effect of PS/Wavelet Detection and Tracking Parameters on Measured Population Dynamics).

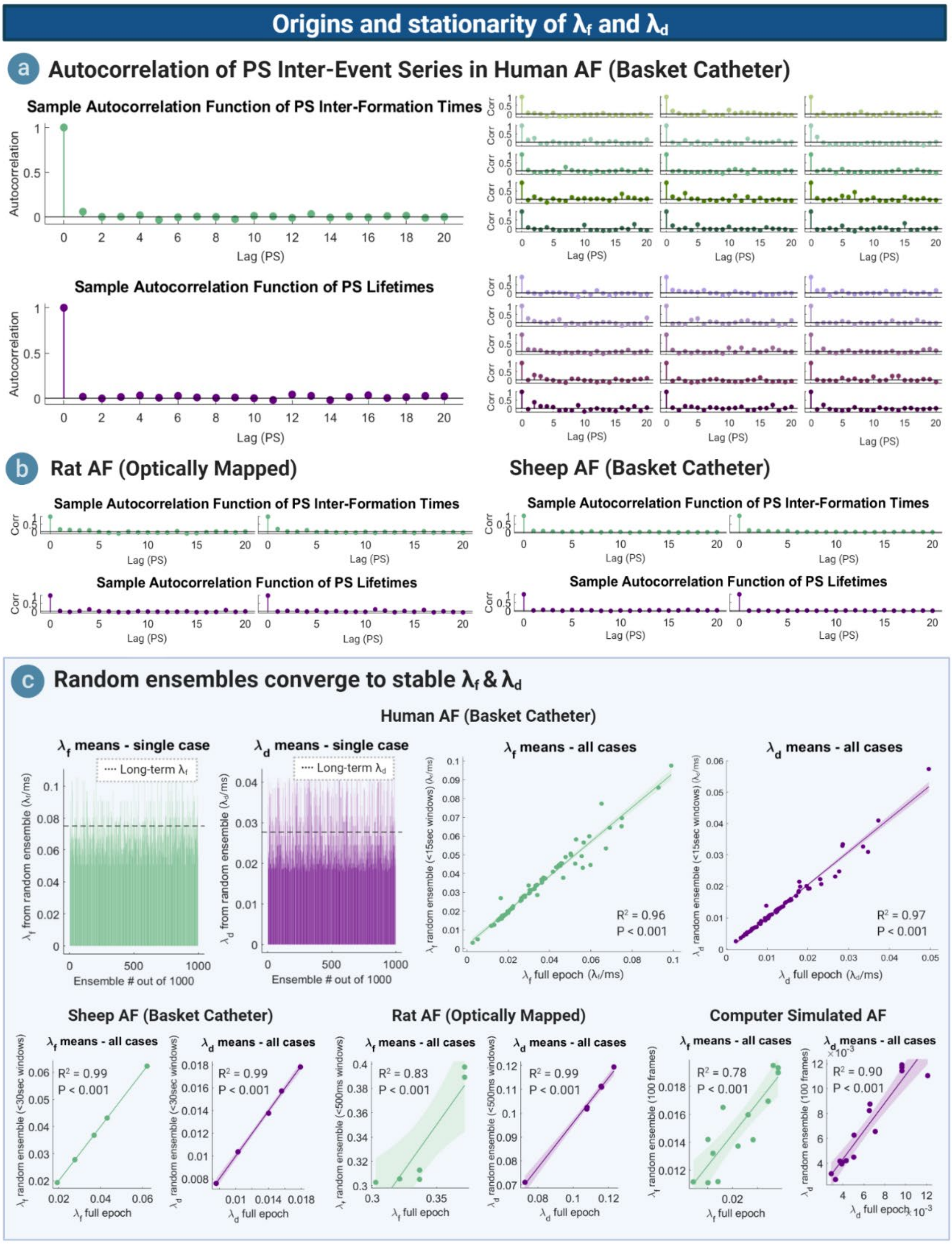

Supplemental Figure 9: Origins and stationarity of λf and λd

(S**7A**) Autocorrelation of PS lifetimes and inter-formation times recorded in all cases of basket catheter mapped human AF approach zero for all non-zero lags, indicating that PS formation/destruction is independent of time. (**S7B**) Autocorrelation of PS lifetimes and inter-formation times in optically mapped rat AF and basket mapped sheep AF also approach zero for all non-zero lags. (**S7C**) Bar plots (top left) depict the running λ_f and λ_d for 1000 random ensembles, which approach the long-term mean λ_f and λ_d. Scatter plots show that in all cases/model systems, λ_f and λ_d measured from short duration random ensembles are highly correlated to λ_f/λ_d from full epoch.

Sample Autocorrelation Function - All Cases

Basket Human AF- Correlograms of PS Inter-Formation Times

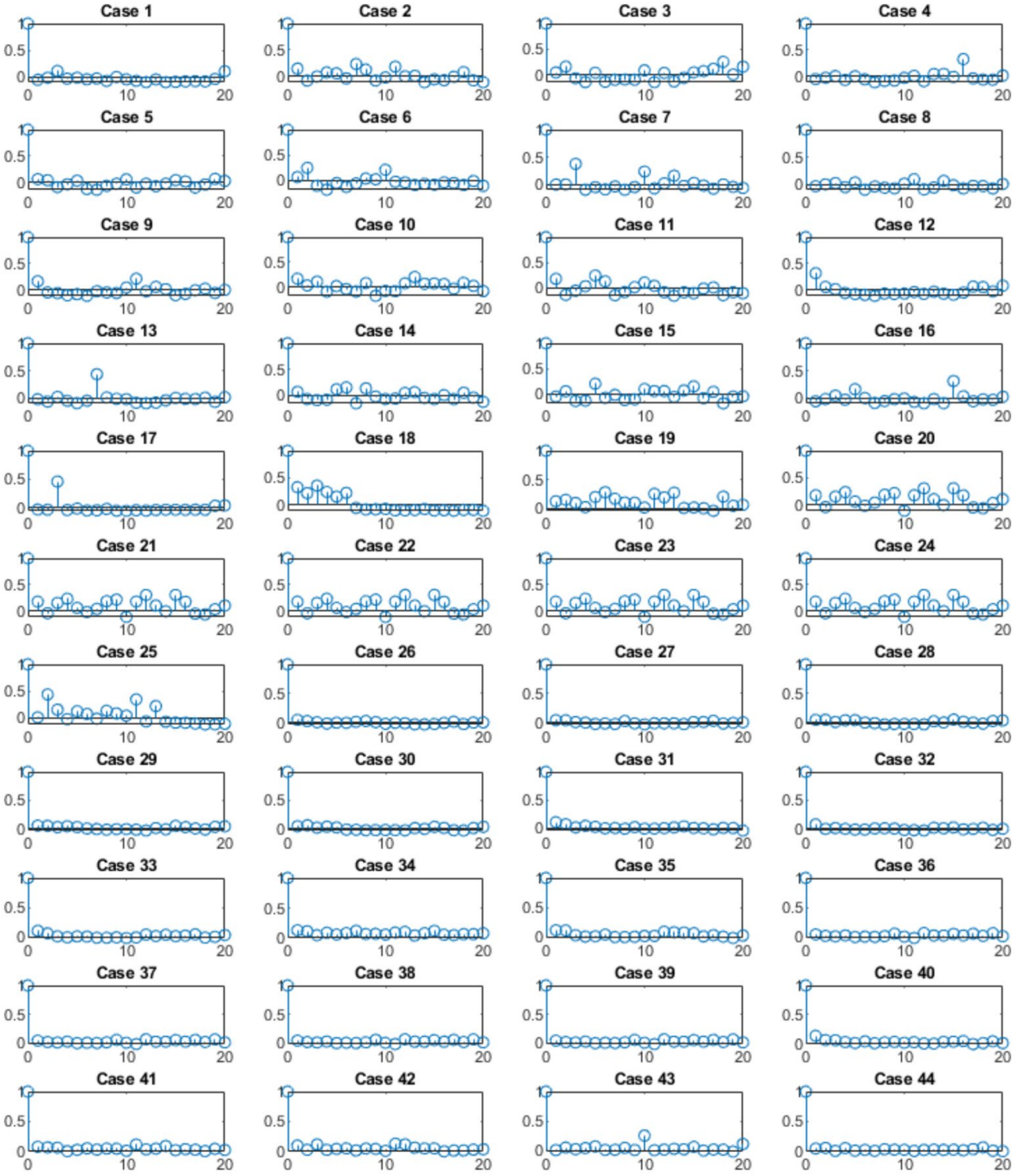

Supplemental Figure 10

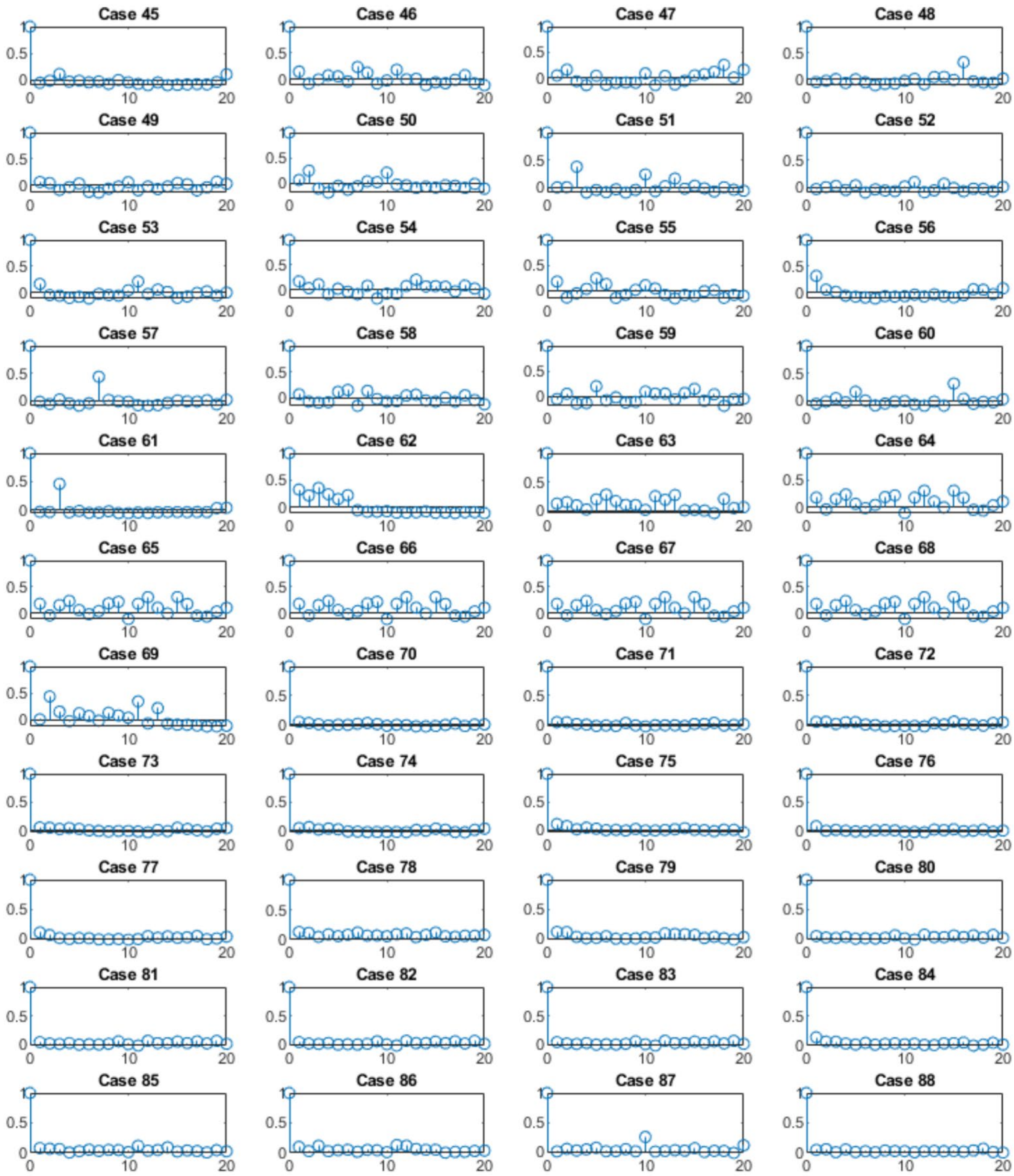

Supplemental Figure 11

Basket Human AF- Correlograms of PS Lifetimes

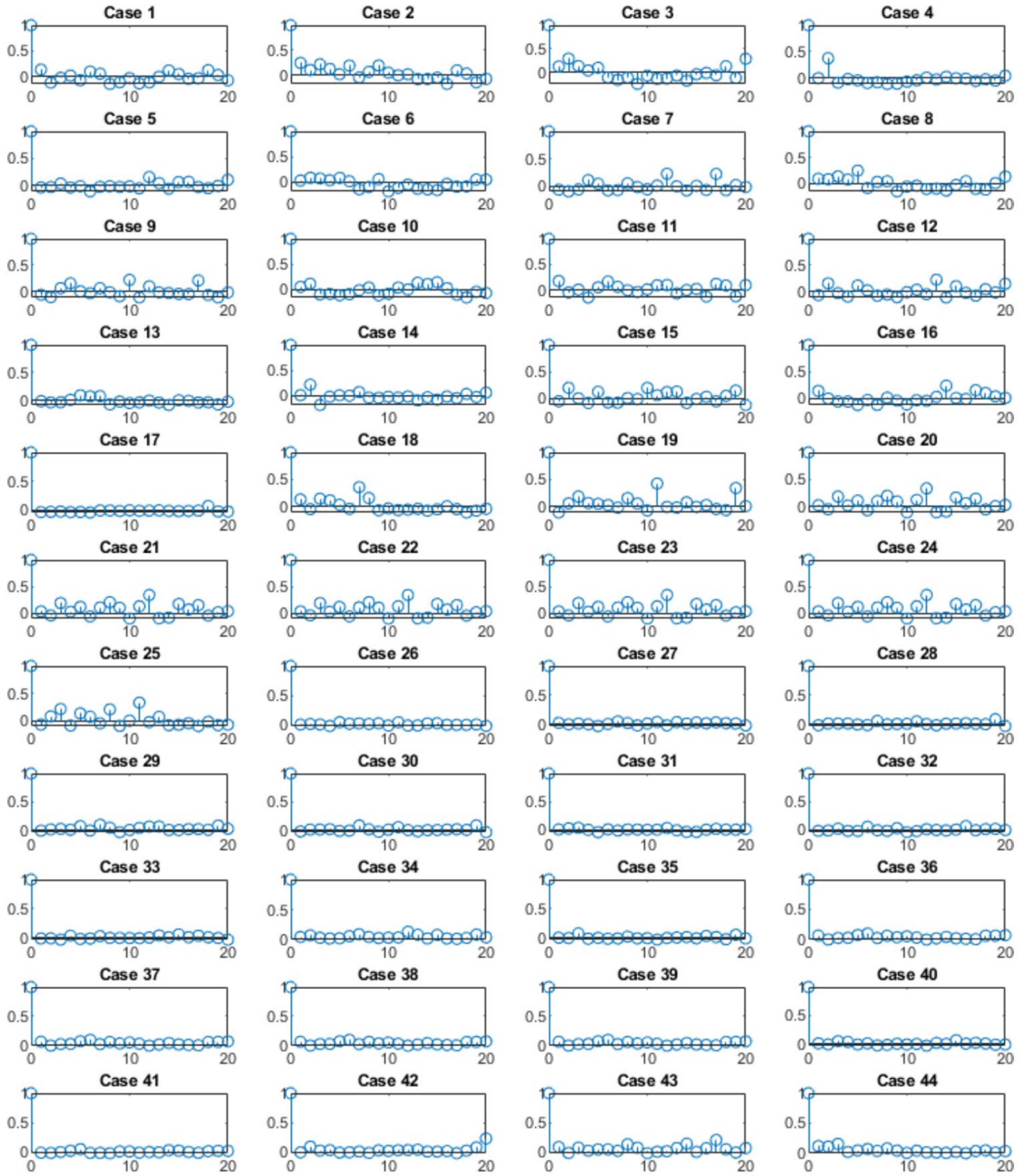

Supplemental Figure 12

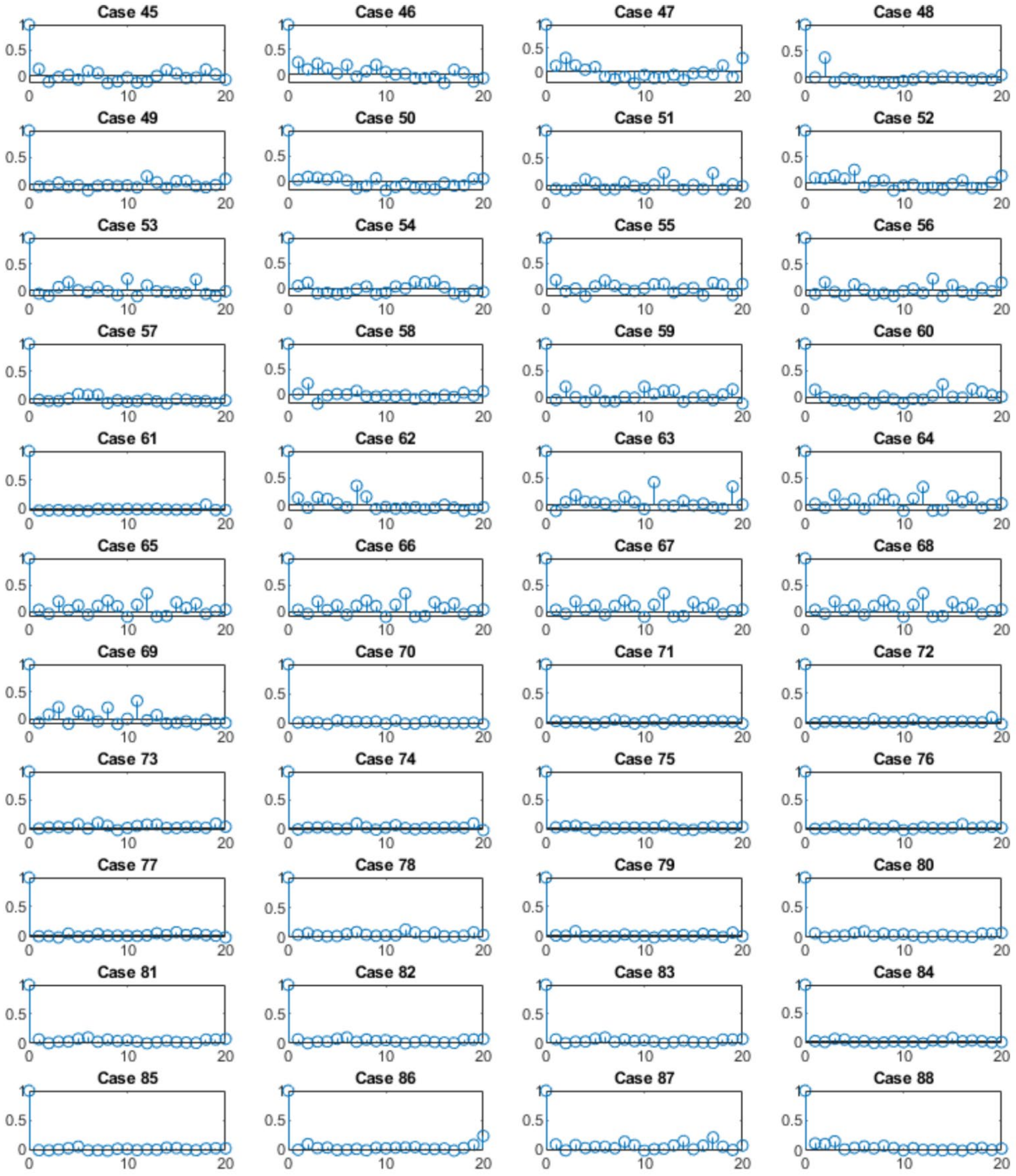

Supplemental Figure 13

HD-Grid Human AF- Correlograms of PS Inter-Formation Times

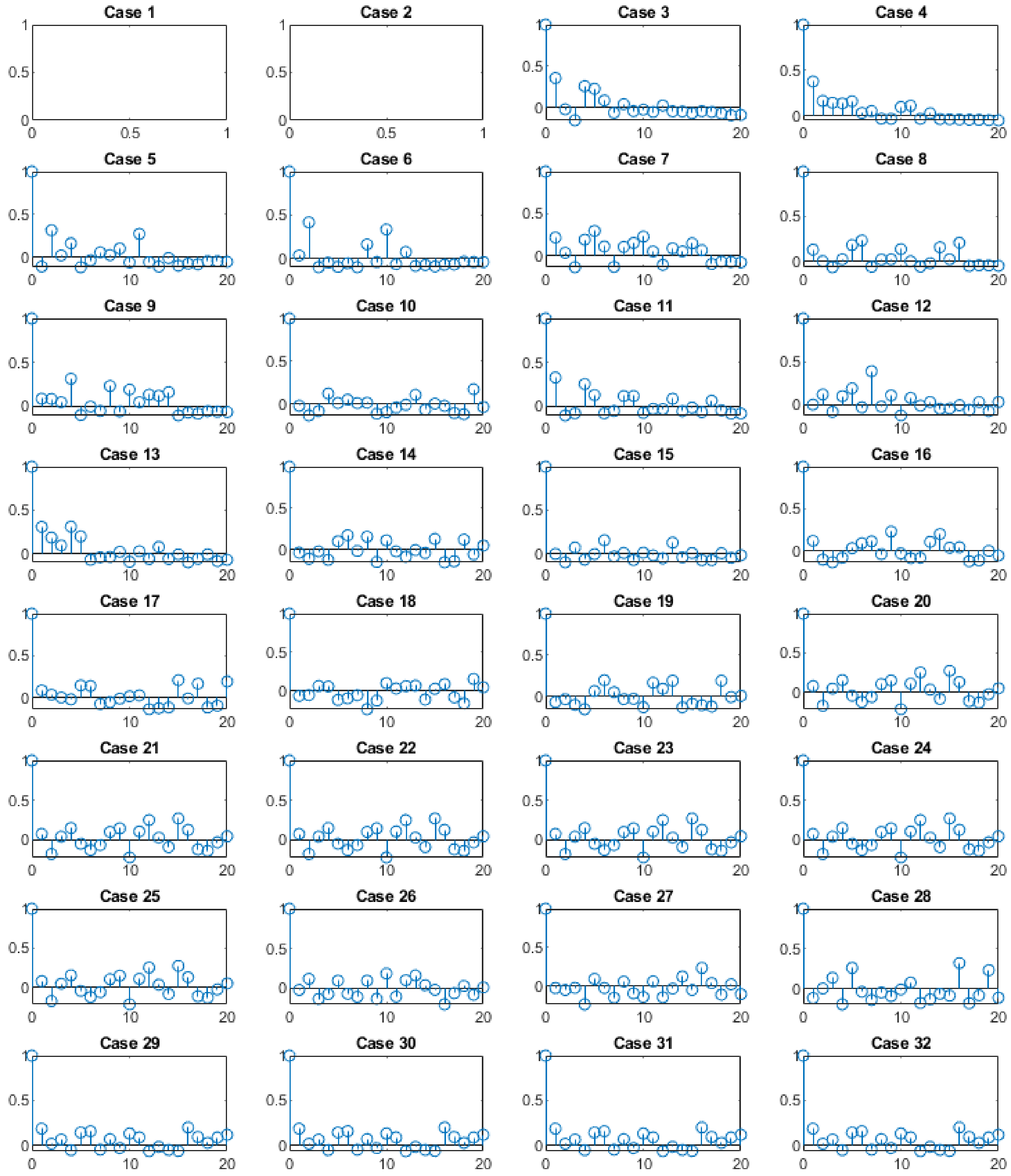

Supplemental Figure 14

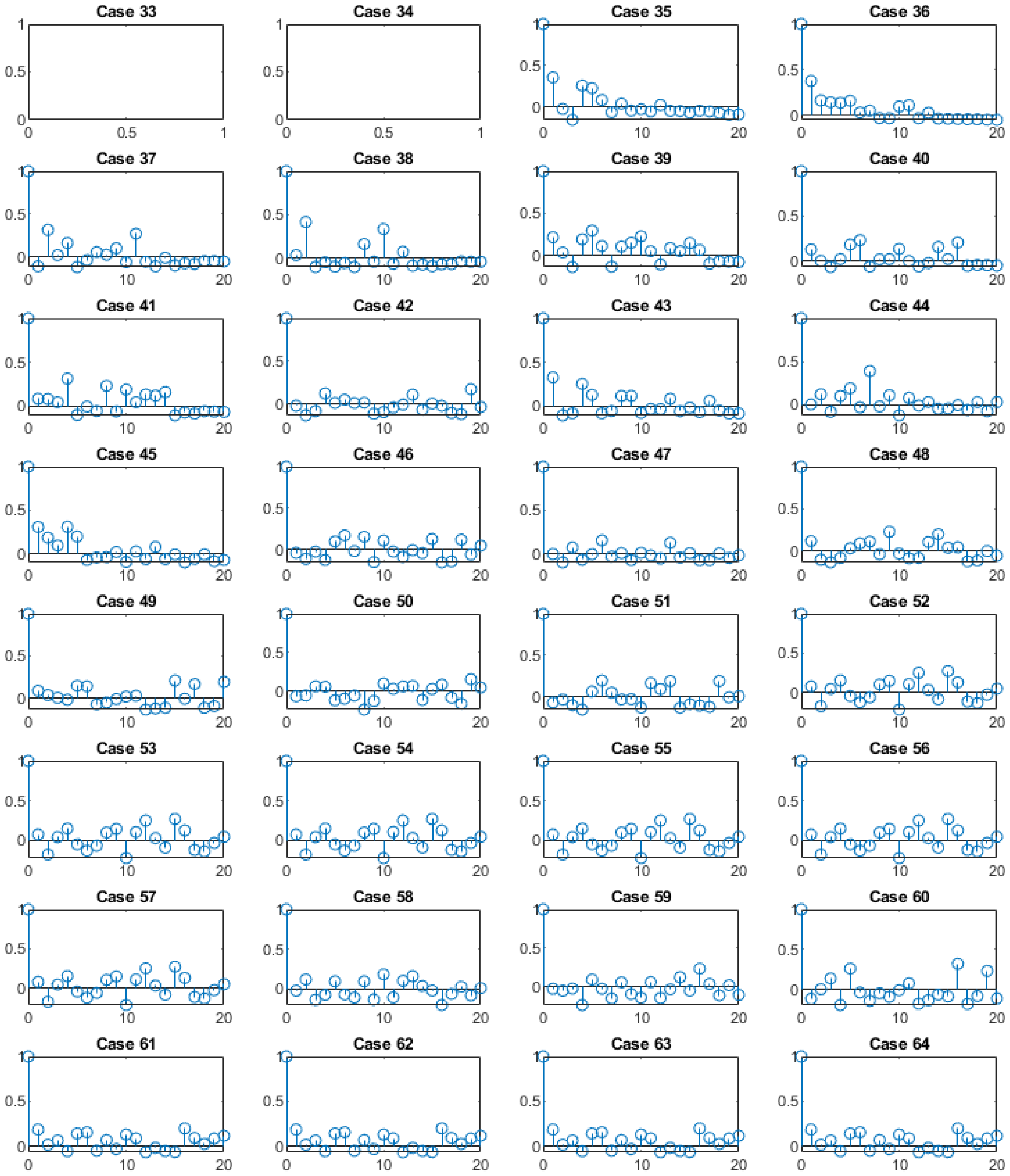

Supplemental Figure 15

HD-Grid Human AF- Correlograms of PS Lifetimes

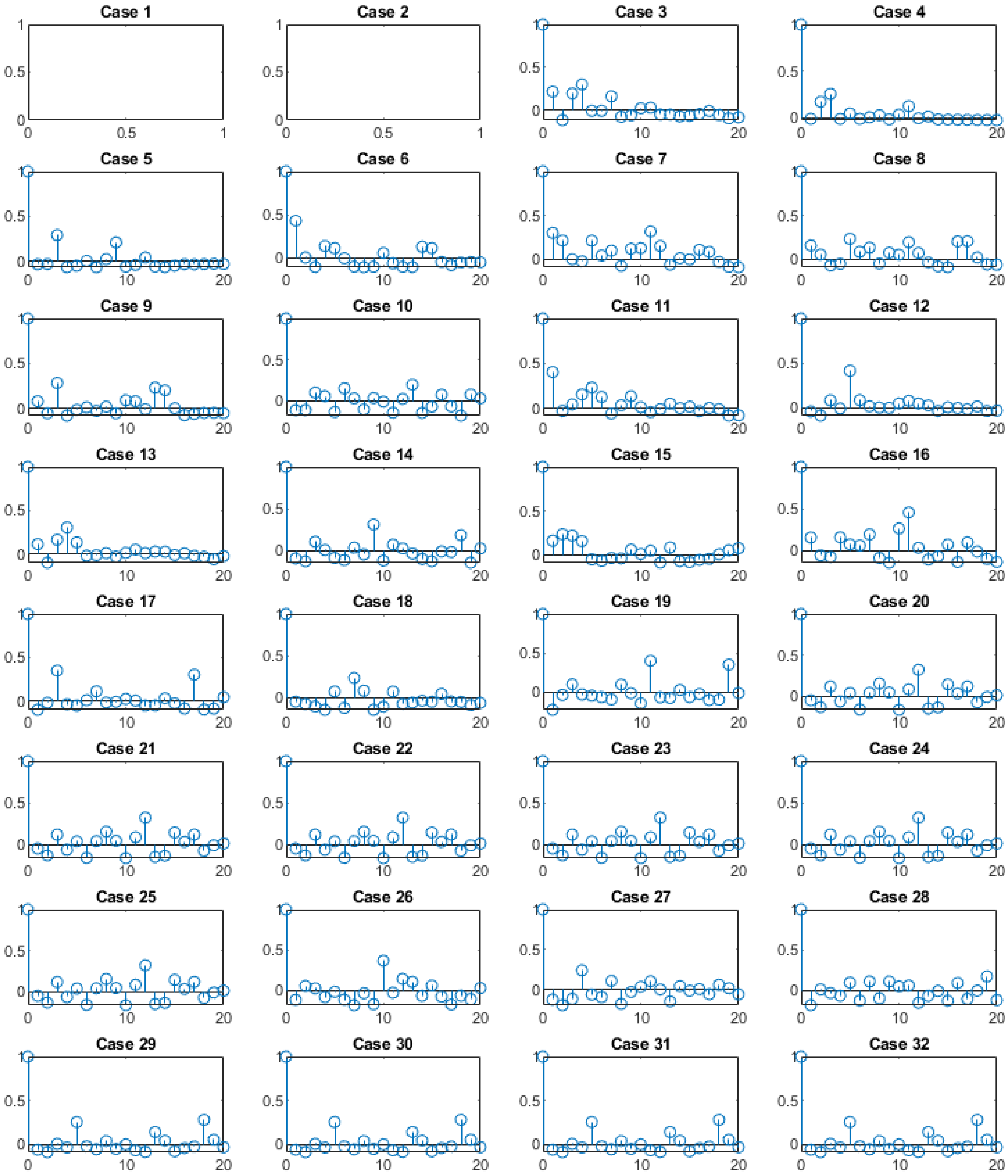

Supplemental Figure 16

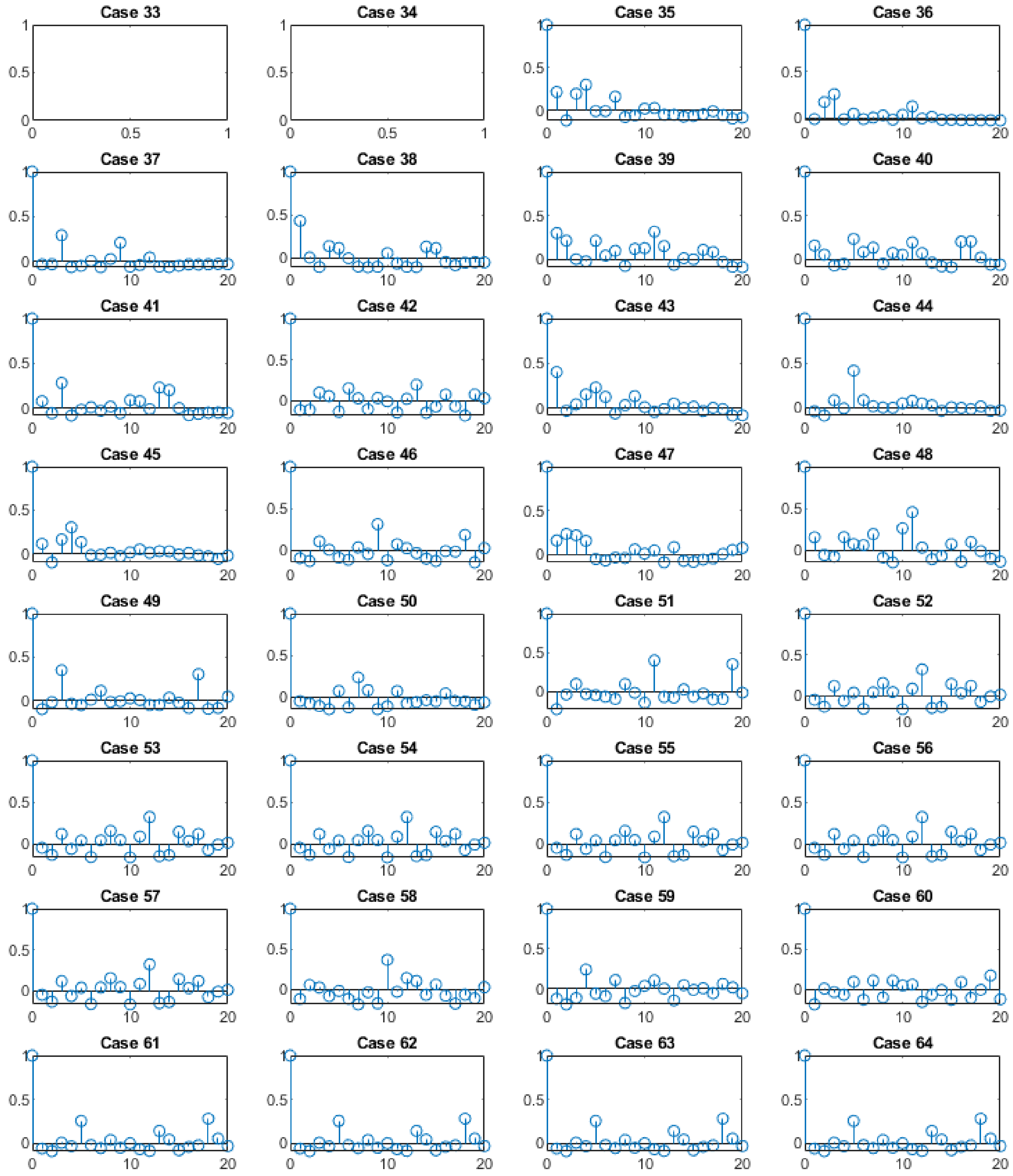

Supplemental Figure 17

Basket Sheep AF- Correlograms of PS Inter-Formation Times

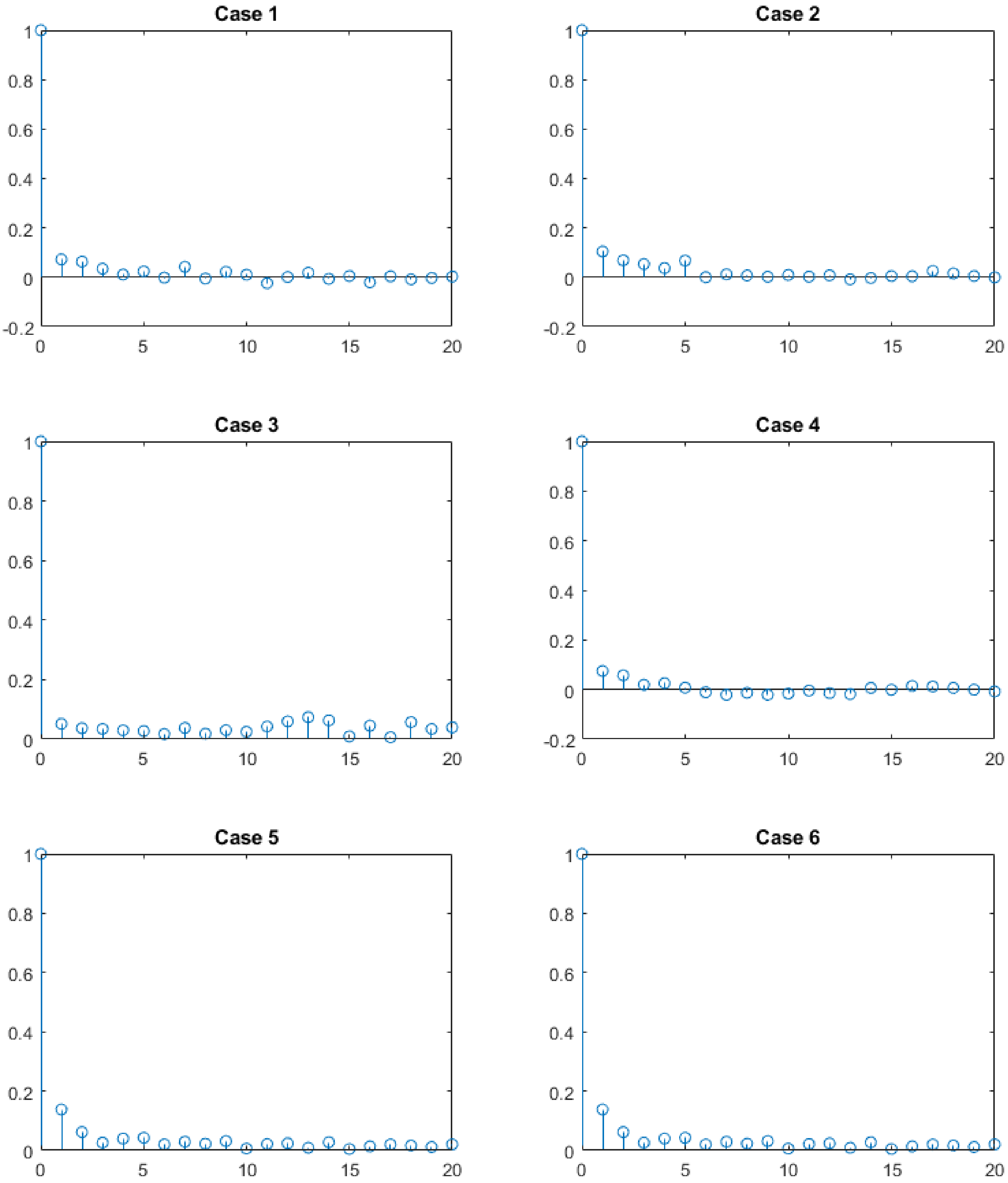

Supplemental Figure 18

Basket Sheep AF- Correlograms of PS Lifetimes Times

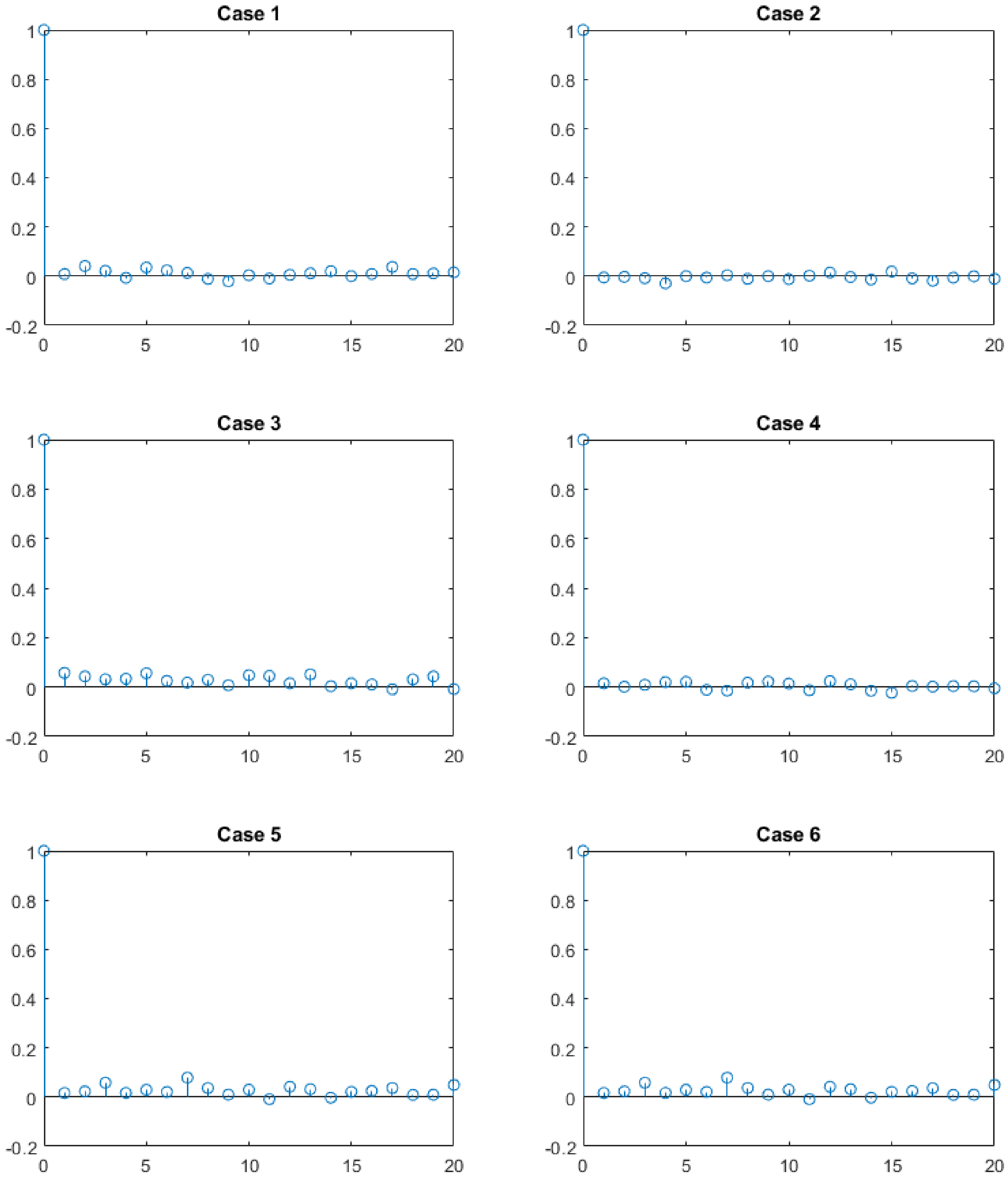

Supplemental Figure 19

Optically Mapped Rat AF- Correlograms of PS Inter-Formation Times

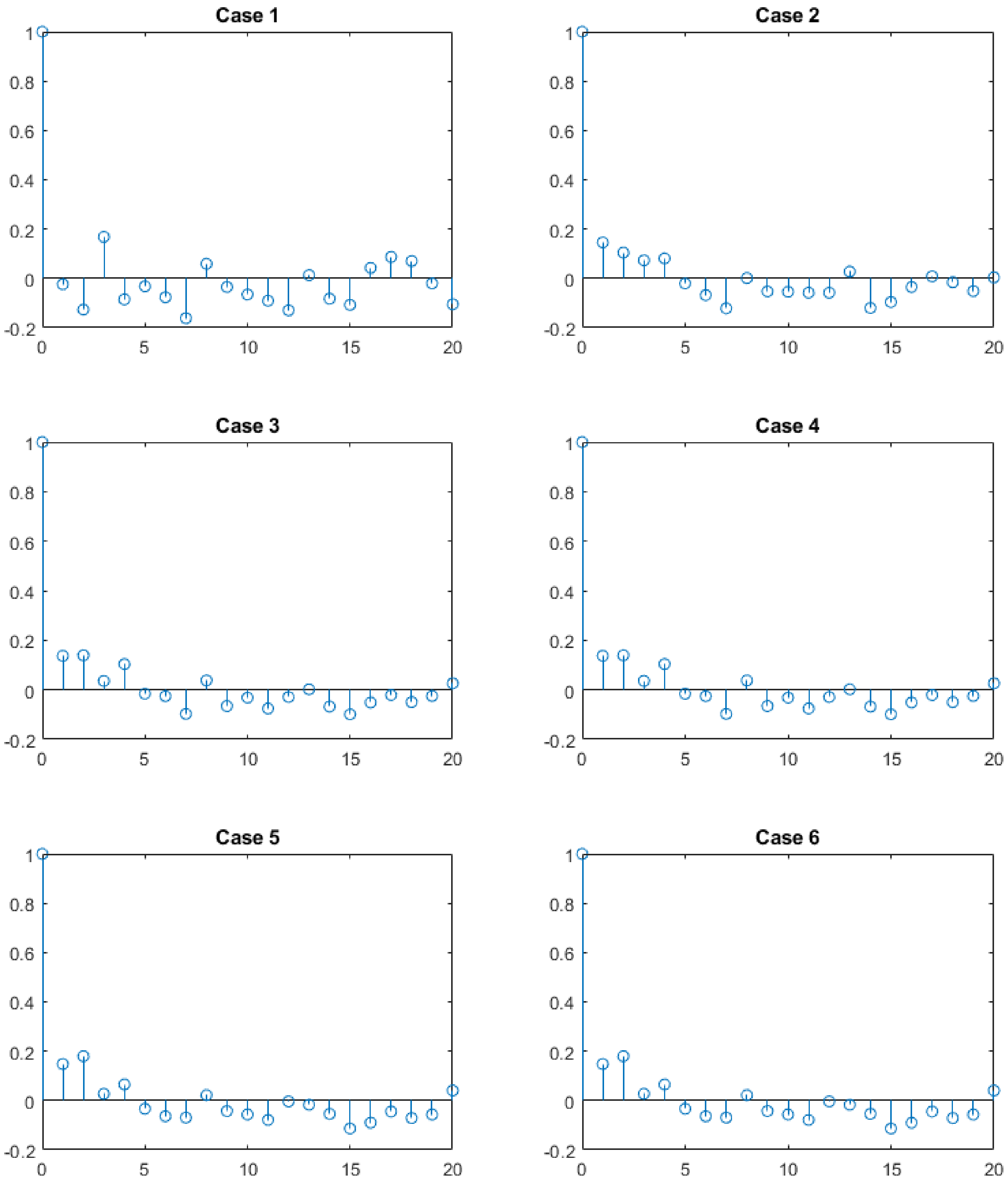

Supplemental Figure 20

Optically Mapped Rat AF- Correlograms of PS Lifetimes

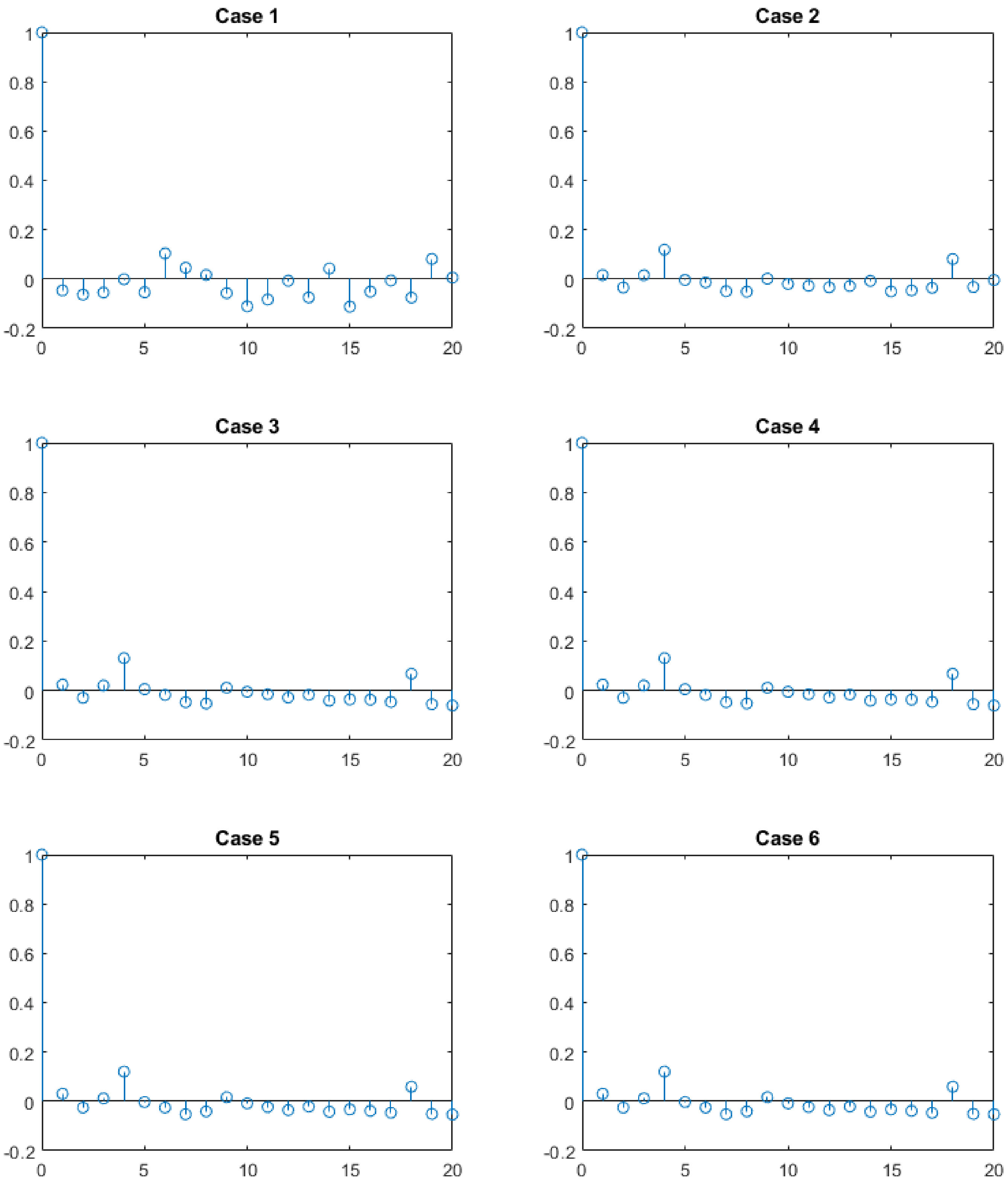

Supplemental Figure 21

Coefficient of variation of λ_f and λ_d

To further validate the stability of λ_f and $\lambda_{d,}$ the coefficient of variation (CV) of λ_f and λ_d was calculated as the ratio of the standard deviation to the mean (SD/mean). For comparison to established clinical measures, CV for dominant frequency (DF) and the AF cycle length (AFCL) was also calculated. AF cycle length was measured using manually electronic calipers to assess AF cycle length on bipolar electrogram data for 20 second intervals over 5 minutes, with AF cycle length determines the mean of AF cycle length interval. Dominant frequency (DF) analysis was performed as described [19]. The regularity index (RI) at each DF was obtained, and only DF possessing an RI>0.2 were included in analyses. DF was computed over 20 second intervals over 5 minutes λ_f and λ_d measurements were computed via measurement over 20 second intervals over 5 minutes.

Coefficient of variation was lower for λ_f (3.7% (95%CI,1.6,5.9)) and λ_d (2.6% (1.5,3.8)) versus DF (16.3% (95%CI,2.5,30)) and AFCL (12.2% (95%CI,6.7,17.7)). Coefficient of variation of λ_f and λ_d was also significantly different to DF ($P\lambda_f < 0.001$; $P\lambda_d < 0.001$) and AFCL ($P\lambda_f < 0.001$; $P\lambda_d < 0.001$).

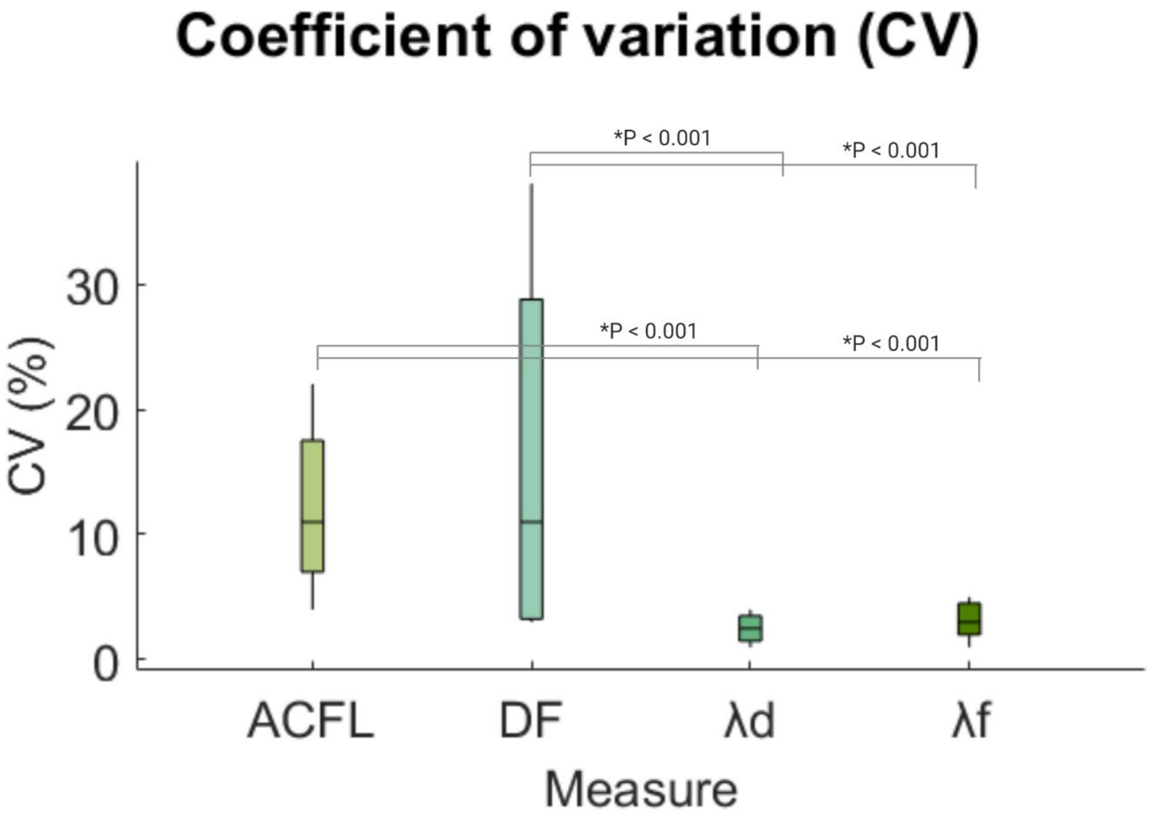

Supplemental Figure 22

S16 Validation using a secondary PS detection algorithm

As an additional cross-validation step, a secondary PS detection algorithm was implemented as described previously by Iyer and Gray.[24].

As shown in Supplemental Figure 23A and E, the PS detected by the convolution kernel and extended topological charge (double ring) methods correspond to the same underlying activity. However, as the convolution kernel method adds the additional step of removing spurious PS not attached to any wavefronts and PS that are attached to the same wavefront, less PS are recorded overall. This results in a lower number of PS detections for the convolution kernel (mean PS number 1.67 PS/frame (95%CI 1.58, 1.74)) versus double ring (mean PS number 3.58 PS/frame (95%CI 2.84, 4.32)) detection algorithm.

Although this affects the measured λ_f and λ_d, PS inter-formation times and lifetimes also produce exponential distributions consistent with a Poisson renewal process using the double ring method (Supplemental Figure 23B and F). This also gives rise to stable rates for λ_f and λ_d as shown by the zero autocorrelation of the PS lifetime inter-event series at all non-zero lags (Supplemental Figure 23C and G).

The double ring PS detection method also showed that PS number and population distribution could be accurately summarised and explained using the M/M/∞ Markov birth-death equation, with computed and observed PS number showing strong correlation (R^2 = 0.85; P < 0.001) (Supplemental Figure 23H). The computed and PS population distribution also showed a high goodness of fit to the observed PS population distribution (χ^2(13, N = 101) P = 0.99) (Supplemental Figure 23H). Collectively, these results are consistent to those seen using the convolution kernel PS detection method.

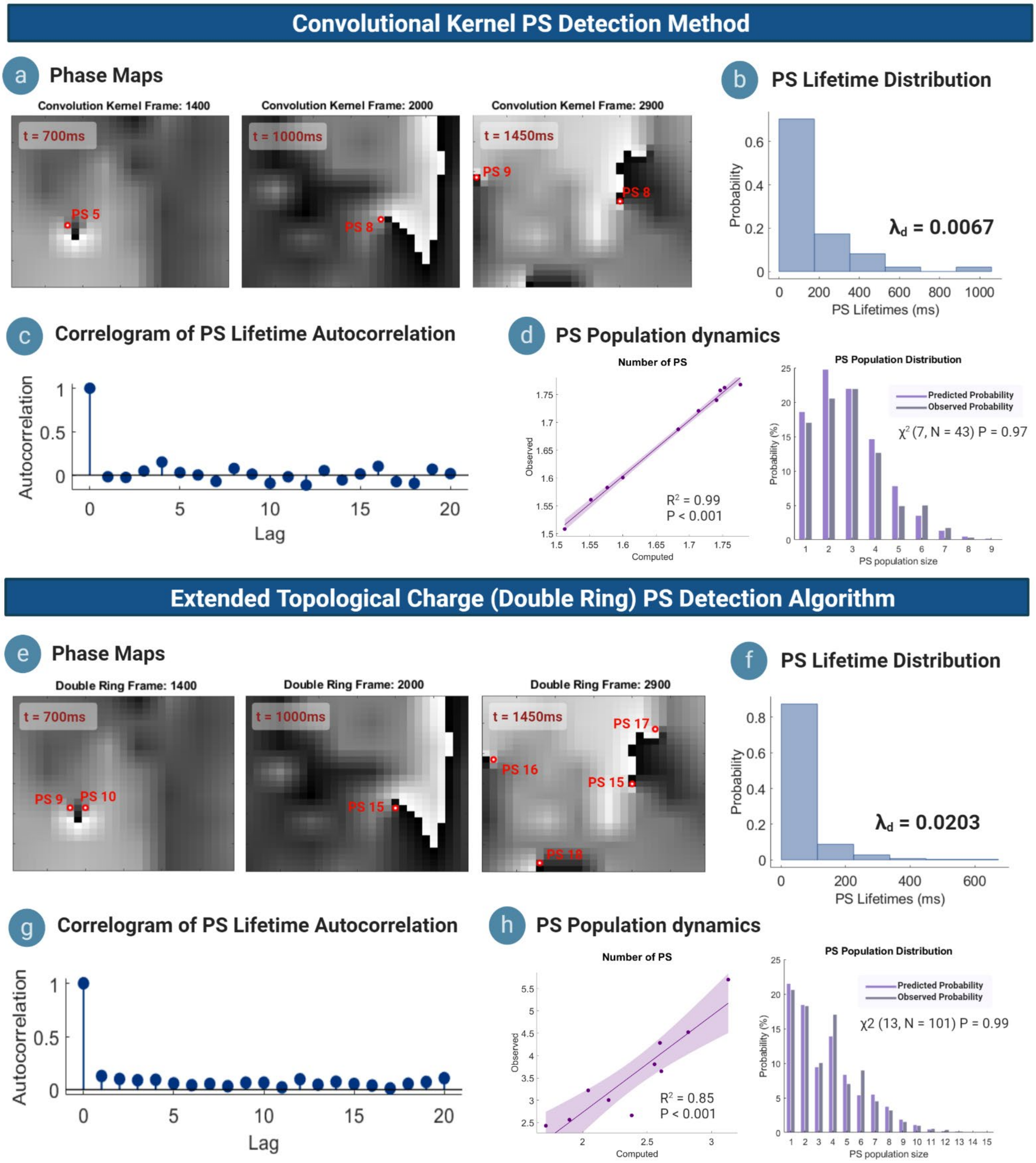

Supplemental Figure 23

S17 Stationarity and Population Dynamics for Long Lasting PS and Wavelets

To evaluate whether results would remain valid when discarding short lasting PS and only looking at events likely representing more stable re-entry, we investigated whether long lasting PS would also show stationarity and follow a Poisson renewal process. To do this, PS > 150ms were pooled across patients and examined.

As shown in the Supplemental Figure 24A and B, longer lasting PS also produced an exponential distribution for PS lifetimes and inter-formation times, with λ_d = 0.023 and λ_f = 0.031 respectively. The exponential distribution of PS lifetimes supports the notion that a Poisson renewal parameter determines the instantaneous probability of destruction of individual PS, that is not related to the lifetime of the PS.

Furthermore, the lifetimes of long-lasting PS show autocorrelation approaching zero at non-zero lags. An example case is shown in Supplemental Figure 24B and D, which plots the lifetimes and inter-formation times for only longer lasting PS present for > 150ms. This further suggests that the instantaneous probability of destruction and formation of individual PS is not related to the lifetime or inter-formation time of the individual PS.

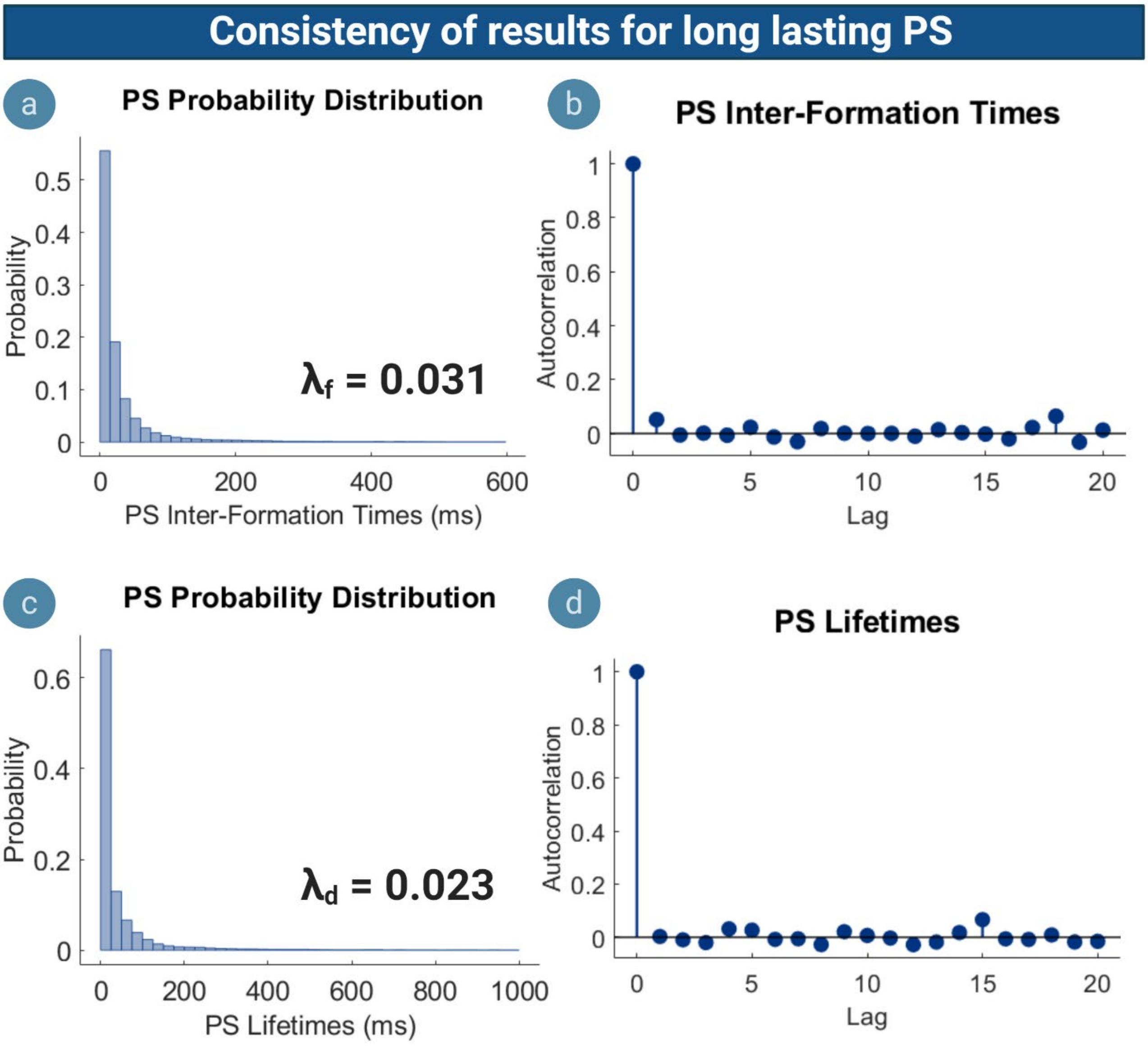

Supplemental Figure 24

1. Aguilar M, Feng J, Vigmond E, Comtois P and Nattel S. Rate-Dependent Role of I(Kur) in Human Atrial Repolarization and Atrial Fibrillation Maintenance. *Biophys J*. 2017;112:1997-2010.
2. Courtemanche M, Ramirez RJ and Nattel S. Ionic mechanisms underlying human atrial action potential properties: insights from a mathematical model. *Am J Physiol*. 1998;275:H301-21.
3. Kneller J, Zou R, Vigmond EJ, Wang Z, Leon LJ and Nattel S. Cholinergic atrial fibrillation in a computer model of a two-dimensional sheet of canine atrial cells with realistic ionic properties. *Circ Res*. 2002;90:E73-87.
4. Dharmaprani D, McGavigan AD, ... and Ganesan AN. Temporal stability and specificity of high bipolar electrogram entropy regions in sustained atrial fibrillation. *J Electrocardiol*. 2019;53:18-27.
5. Dharmaprani D, Schopp M, Kuklik P, Chapman D, Lahiri A, Dykes L, Xiong F, Aguilar M, Strauss B, Mitchell L, Pope K, Meyer C, Willems S, Akar FG, Nattel S, McGavigan AD and Ganesan AN. Renewal Theory as a Universal Quantitative Framework to Characterize Phase Singularity Regeneration in Mammalian Cardiac Fibrillation. *Circ Arrhythm Electrophysiol*. 2019;12:e007569.
6. Hiram R, Naud P, Xiong F, Al-U'datt D, Algalarrondo V, Sirois MG, Tanguay JF, Tardif JC and Nattel S. Right Atrial Mechanisms of Atrial Fibrillation in a Rat Model of Right Heart Disease. *J Am Coll Cardiol*. 2019;74:1332-1347.
7. Shkurovich S, Sahakian AV and Swiryn S. Detection of atrial activity from high-voltage leads of implantable ventricular defibrillators using a cancellation technique. *IEEE Trans Biomed Eng*. 1998;45:229-34.
8. Pan J and Tompkins WJ. A real-time QRS detection algorithm. *IEEE Trans Biomed Eng*. 1985;32:230-6.
9. Roney CH, Cantwell CD, Qureshi NA, Chowdhury RA, Dupont E, Lim PB, Vigmond EJ, Tweedy JH, Ng FS and Peters NS. Rotor Tracking Using Phase of Electrograms Recorded During Atrial Fibrillation. *Ann Biomed Eng*. 2017;45:910-923.
10. Bray MA and Wikswo JP. Considerations in phase plane analysis for nonstationary reentrant cardiac behavior. *Phys Rev E Stat Nonlin Soft Matter Phys*. 2002;65:051902.
11. Kuklik P, Zeemering S, Maesen B, Maessen J, Crijns HJ, Verheule S, Ganesan AN and Schotten U. Reconstruction of instantaneous phase of unipolar atrial contact electrogram using a concept of sinusoidal recomposition and Hilbert transform. *IEEE Trans Biomed Eng*. 2015;62:296-302.
12. Vidmar D and Rappel WJ. To the Editor- On the deformation and interpolation of phase maps. *Heart Rhythm*. 2018;15:e3.
13. Nash MP, Mourad A, Clayton RH, Sutton PM, Bradley CP, Hayward M, Paterson DJ and Taggart P. Evidence for multiple mechanisms in human ventricular fibrillation. *Circulation*. 2006;114:536-42.
14. Kuklik P, Zeemering S, van Hunnik A, Maesen B, Pison L, Lau DH, Maessen J, Podziemski P, Meyer C, Schaffer B, Crijns H, Willems S and Schotten U. Identification of Rotors during Human Atrial Fibrillation Using Contact Mapping and Phase Singularity Detection: Technical Considerations. *IEEE Trans Biomed Eng*. 2017;64:310-318.
15. Clayton RH and Nash MP. Analysis of cardiac fibrillation using phase mapping. *Card Electrophysiol Clin*. 2015;7:49-58.
16. Rogers JM. Combined phase singularity and wavefront analysis for optical maps of ventricular fibrillation. *IEEE Trans Biomed Eng*. 2004;51:56-65.
17. Garfinkel A, Chen PS, Walter DO, Karagueuzian HS, Kogan B, Evans SJ, Karpoukhin M, Hwang C, Uchida T, Gotoh M, Nwasokwa O, Sager P and Weiss JN. Quasiperiodicity and chaos in cardiac fibrillation. *J Clin Invest*. 1997;99:305-14.
18. Gallager RG. *Discrete stochastic processes*: Springer Science & Business Media; 2012.
19. Sanders P, Berenfeld O, Hocini M, Jais P, Vaidyanathan R, Hsu LF, Garrigue S, Takahashi Y, Rotter M, Sacher F, Scavee C, Ploutz-Snyder R, Jalife J and Haissaguerre M. Spectral analysis identifies sites of high-frequency activity maintaining atrial fibrillation in humans. *Circulation*. 2005;112:789-97.
20. Oppenheim AV and Verghese GC. *Signals, systems and inference*: Pearson; 2015.
21. Novozhilov AS, Karev GP and Koonin EV. Biological applications of the theory of birth-and-death processes. *Briefings in Bioinformatics*. 2006;7:70-85.
22. Crawford FW, Ho LST and Suchard MA. Computational methods for birth-death processes. *Wiley interdisciplinary reviews Computational statistics*. 2018;10.
23. Boyd S, Diaconis P and Xiao L. Fastest mixing Markov chain on a graph. *SIAM review*. 2004;46:667-689.
24. Iyer AN and Gray RA. An experimentalist's approach to accurate localization of phase singularities during reentry. *Ann Biomed Eng*. 2001;29:47-59.
